# Toward azo-linked covalent organic frameworks by developing linkage chemistry via linker exchange

**DOI:** 10.1038/s41467-022-29814-3

**Published:** 2022-04-21

**Authors:** Zhi-Bei Zhou, Peng-Ju Tian, Jin Yao, Ya Lu, Qiao-Yan Qi, Xin Zhao

**Affiliations:** Key Laboratory of Synthetic and Self-Assembly Chemistry for Organic Functional Molecules, Center for Excellence in Molecular Synthesis, Shanghai Institute of Organic Chemistry, University of Chinese Academy of Sciences, Chinese Academy of Sciences, Shanghai, China

**Keywords:** Polymer synthesis, Polymers, Polymers

## Abstract

Exploring new linkage chemistry for covalent organic frameworks (COFs) provides a strong driving force to promote the development of this emerging class of crystalline porous organic materials. Herein we report a strategy to synthesize COFs with azo linkage, one of the most important functional unit in materials science but having not yet been exploited as a linkage of COFs. This strategy is developed on the basis of in situ linker exchange, by which imine-linked COFs are completely transformed into azo-linked COFs (Azo-COFs). Moreover, distinct properties of Azo-COFs from their corresponding imine-linked precursors are observed, indicating unique property of Azo-COFs. This strategy provides a useful approach to develop new linkage chemistry for COFs. It also has established a synthetic method for azo-linked COFs, which not only enriches the family of COFs but also offers a platform to explore properties and applications of this class of crystalline porous conjugated polymers.

## Abstract (web summary)

Exploring new linkage chemistry for covalent organic frameworks (COFs) provides a strong driving force to promote the development of this class porous materials. Here, the authors report a strategy to synthesize COFs with azo linkages based on an in situ linker exchange strategy which transforms imine-linked COFs into their azo-linked counterparts, and explore the unique properties of azo-linked COFs.

## Introduction

Covalent organic frameworks (COFs) are an emerging class of crystalline porous organic materials assembled by covalently linking building blocks in sequence^1–3^. Benefiting from their unique structural features, COFs have achieved versatile applications in many fields such as gas storage, separation, catalysis, optoelectronics, sensing, drug delivery, electrochemical applications, etc^4–6^. During the development history of COFs, exploring new linkage chemistry has always been one of research focuses, for the emergence of a new type of linkage not only enriches the structural diversity of COFs but more importantly could bring in distinctive properties and applications. Over the past dozen years, COFs with –B–O–^7,8^, –C=N–^9,10^, –B–N–^11^, –C=C–^12,13^, –C–N–^14–20^, –C–O–^21–23^, –Si–O–^24^, and –C–C–^25,26^ linkages have been achieved. However, it remains a formidable challenge in developing new linkages for COFs to further explore novel connectivity and functionality.

Azo unit (–N=N–), as a vital functional group in chemistry and materials science, has aroused wide interest because of its intriguing features such as photo-isomerization^27–29^, narrow bandgap^30^, Lewis base property^31^, and redox activity^32^. In this context, construction of azo-linked COFs (Azo-COFs) undoubtedly will lead to a novel type of crystalline porous polymers with fascinating properties. Indeed, recent theoretical calculations have revealed that Azo-COFs have great potentials in photocatalysis^33^. However, thus far –N=N– has not yet been reported as a linkage to construct COFs, albeit a few COFs carrying azo moieties were prepared through polycondensation of judiciously selected monomers with pre-installed –N=N– units^32,34–38^. In those examples the azo units do not function as linkages, but as part of building blocks. This might be attributed to lack of an appropriate reaction or condensation condition to implement azo bond into crystalline frameworks. In this contribution, a synthetic method for Azo-COFs has been established, by which –N=N– linkage has been developed for COFs. Moreover, distinct photocatalytic property between an Azo-COF and its corresponding imine-linked COF progenitor is also revealed.

## Results

### Development of the synthetic method for Azo-COFs

To achieve the synthesis of Azo-COFs, we firstly tried Mills reaction which has been widely used to construct azo bond via the reaction of amine and nitrosocompound^39,40^. However, only amorphous products were produced even though various condensation conditions were screened (Supplementary Section 2). This might be attributed to low reversibility of azo bond which is incapable of structure repairing to produce crystalline frameworks under these conditions. To solve this problem, a strategy that can bypass direct polycondensation of monomers is preferred. In 2017 we report a linker-exchange-mediated COF-to-COF transformation strategy, by which an imine-linked COF could be converted into another imine-linked COF with different structure via in situ replacing the linker of the former with a new linker^41^. Such a building block exchange approach has lately been extended to transformation between COFs and even between covalent organic polymers (COPs) and COFs with different linkages^42,43^. However, so far it has been limited to the linkages already established. Whether it could be employed to explore new linkage chemistry for COFs is unclear. We envisioned that the original frameworks could serve as templates to guide the growth of new frameworks in the building block replacement process. As a result, the crystallization problem encountered in the polycondensation of monomers might be overcome. Based on this concept, we changed the synthetic method of Azo-COFs from direct polycondensation of monomers to the linker-exchange strategy. To this end, the experiment was designed to establish azo bond by using 1,4-dinitrosobenzene (DNB) to replace the linker corresponding to terephthalaldehyde (TPA) in imine-linked COFs.

**Fig. 1:**
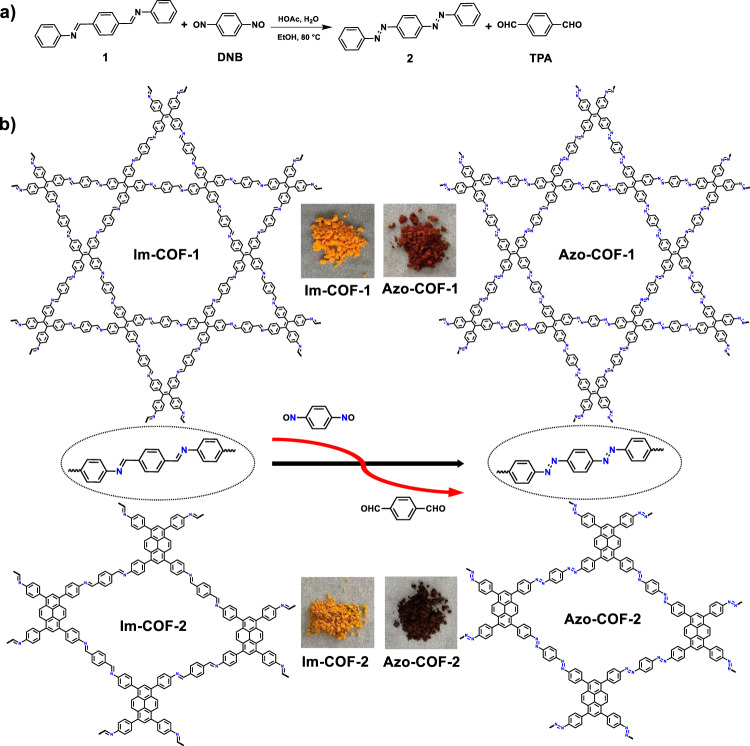
Schematic diagram for the linker-exchange strategy. **a** Model reaction. **b** Fabrication of azo-linked COFs via linker exchange.

We firstly performed a model reaction to verify feasibility of this design (Fig. 1a). The model reaction clearly indicated a complete conversion from the imine precursor to the azo product (Supplementary Section 3). Encouraged by the success of the model reaction, we then conducted a study on the synthesis of azo-linked COFs via the COF-to-COF transformation approach. To our delight, upon treating the imine-linked COFs (Im-COF-1^44,45^ and Im-COF-2^46^) with DNB under solvothermal conditions, azo-linked COFs, termed Azo-COF-1 and Azo-COF-2, respectively, were successfully obtained (Fig. 1b). Their structures were unambiguously elucidated based on a variety of characterization, which is discussed below.

### Characterization of the COF-to-COF transformation

**Fig. 2:**
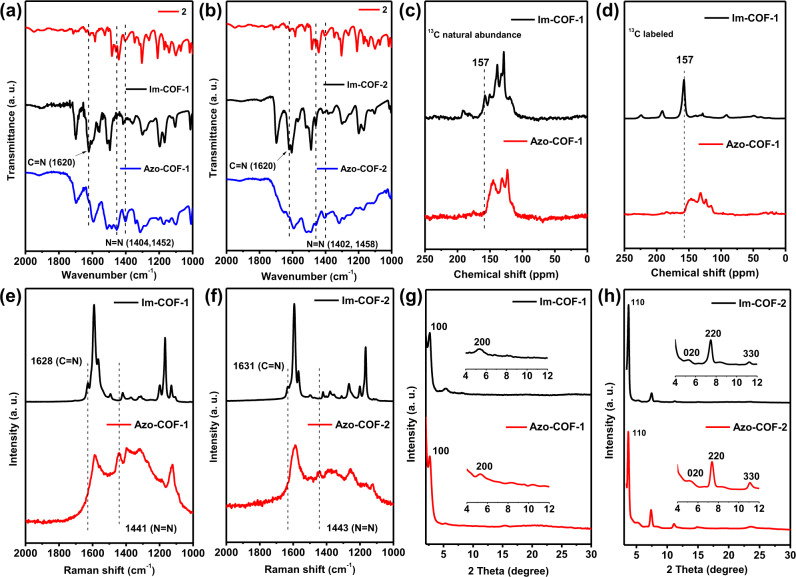
Characterization of the structural transformation. Comparisons of (**a**, **b**) FT-IR spectra, (**c**, **d**) ^13^C CP-MAS NMR spectra, (**e**, **f**) Raman spectra, and (**g**, **h**) PXRD patterns between the imine-linked COFs and azo-linked COFs.

The successful transformation from Im-COF-1 to Azo-COF-1 was revealed by Fourier transform infrared (FT-IR) spectroscopy (Fig. 2a). The band corresponding to –C=N– unit (1620 cm^−1^) disappears in the spectrum of Azo-COF-1. Correspondingly, two peaks belonging to –N=N– moiety (1404 and 1452 cm^−1^) are observed in the same spectrum, suggesting successful construction of azo-linked structure^47^. Similar results are also found for the transformation from Im-COF-2 to Azo-COF-2 (Fig. 2b), as illustrated by the disappearance of the peak corresponding to imine band and emergence of azo peaks after the conversion reaction.

Solid-state ^13^C cross-polarization magic angle spinning (CP-MAS) NMR spectra of the COFs indicate that the carbon signals of –C=N– linkages appear at 157 and 156 ppm for Im-COF-1 and Im-COF-2, respectively (Fig. 2c and Supplementary Section 5). These peaks are not observed in the spectra of the resultant Azo-COFs, indicating that the –C=N– units are completely replaced. To provide stronger evidence for the complete linkage transformation, half of the imine carbon atoms in the Im-COFs were labeled with ^13^C (Supplementary Section 4). The intense peak of ^13^C-labeled imine carbon completely disappeared after the exchange reaction, clearly indicating a full linkage conversion (Fig. 2d and Supplementary Fig. 8).

To collect more evidence for the framework conversions, Raman spectroscopy was performed (Supplementary Section 6). Raman spectra of compounds **1** and **2** of the model reaction were compared, which indicated vanishment of imine (1621 cm^−1^) and emergence of a peak corresponding to azo bond (1432 cm^−1^) after the reaction, suggesting conversion from imine to azo compound. For the transformation from Im-COF-1 to Azo-COF-1, the comparison between their spectra reveals disappearance of imine linkage (1628 cm^−1^) and presence of azo linkage (1441 cm^−1^) in the spectrum of the latter (Fig. 2e), indicating achievement of linkage conversion. Moreover, quite different from that of Im-COF-1, the Raman spectrum of Azo-COF-1 displays broadening peaks due to the background interference of fluorescence^32^, further supporting the occurrence of framework transformation. For Azo-COF-2, similar phenomena such as widening peaks, disappearance of imine peak (1631 cm^−1^), and appearance of azo peak (1443 cm^−1^) were also observed (Fig. 2f).

Crystallinity of the as-formed Azo-COFs was checked with powder X-ray diffraction (PXRD). The comparisons between the PXRD patterns of the Im-COFs and Azo-COFs show high similarity in both cases (Fig. 2g, h), indicating that they have very similar lattice cells. Pawley refinement was conducted to give unit cell parameters of the COFs (Supplementary Section 7). Compared to that of Im-COF-1 (a = b = 38.6435 Å, c = 4.9822 Å), a slight decrease in unit cell was found for Azo-COF-1 (a = b = 38.3586 Å, c = 4.5707 Å). A similar trend was also observed for Azo-COF-2. It could be attributed to the shorter bond length of N=N than that of C=N.

**Fig. 3:**
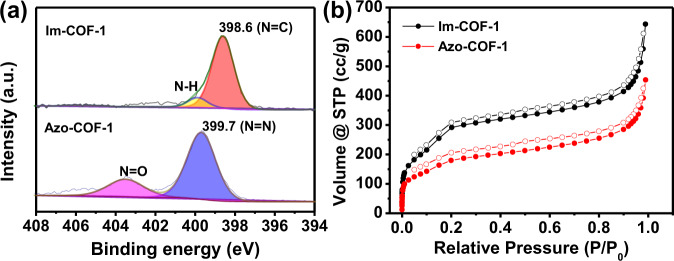
XPS analysis and porosity. Comparisons of (**a**) high-resolution XPS of N 1*s* and (**b**) N_2_ sorption between Im-COF-1 and Azo-COF-1. Note: the supernumerary peak at 403.4 eV in the XPS spectrum of Azo-COF-1 was attributed to the N=O moieties resulting from the adsorbed monomer and residual groups^51^.

X-ray photoelectron spectroscopy (XPS) provides another evidence for the transformation from imine-linked COFs to azo-linked COFs (Supplementary Section 8). The comparison of their XPS spectra indicated that, after the exchange reaction, the binding energy of N 1*s* shifted from 398.6 eV of Im-COF-1 to 399.7 eV of Azo-COF-1 (Fig. 3a), suggesting a conversion from imine linkage to azo linkage^48,49^. In the case of Azo-COF-2, the same shift of N 1*s* peak was also observed, which is in line with the linkage transformation.

N_2_ sorption experiment was applied for the COFs to assess their porosity (Fig. 3b and Supplementary Section 9). On the basis of the sorption data, the Brunauer–Emmett–Teller surface area of Azo-COF-1 was calculated to be 533 m^2^/g, while a value of 738 m^2^/g was obtained for Azo-COF-2. Compared with those of the Imine COF precursors, the surface areas of the Azo-linked COFs decrease. This might be attributed to a decrease in crystallinity after the conversion and the trapped monomer molecules in the pore of the Azo-COFs. Pore size distribution analysis revealed that Azo-COF-1 exhibited two distributions around 9.5 and 29.8 Å, respectively, in consistent with the predicted dual-pore structure. For Azo-COF-2, a narrow peak at 19.8 Å was observed, which well matches with aperture size of the theoretically simulated structure of Azo-COF-2. In both cases, slight decrease in the aperture sizes was observed in comparison to the imine-linked COF precursors, which might be attributed to the smaller unit cells of the Azo-COFs and the presence of adsorbed monomers within the pores.

Scanning electron microscopy (SEM) was employed to reveal morphology of the COFs before and after the transformation. For Im-COF-1 and Azo-COF-1, similar morphology was identified by their SEM images, which showed amalgamation of flakes. However, Azo-COF-1 exhibited a smaller size domain of flakes compared with its maternal Im-COF-1. A remarkable difference in morphology was observed for Im-COF-2 and Azo-COF-2, wherein the former had a granular-aggregation morphology and the latter displayed a ribbon-like structure (Supplementary Section 10).

### Hydrolysis experiments

**Fig. 4:**
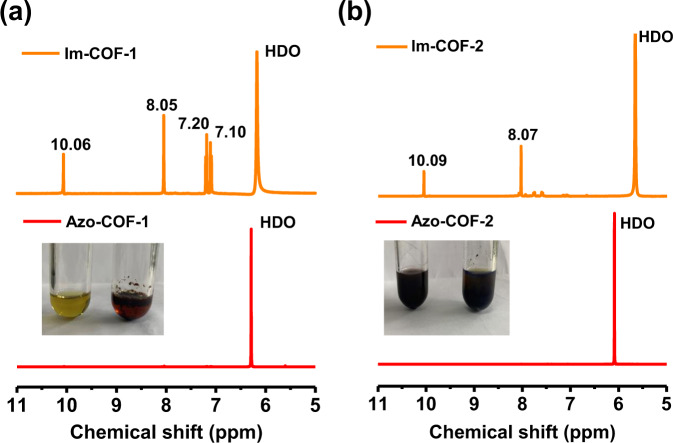
Results of the hydrolysis experiments. ^1^H NMR spectra of (**a**) Im-COF-1 and Azo-COF-1, and (**b**) Im-COF-2 and Azo-COF-2 after being treated with DCl in DMSO-*d*_6_. Insets: photographs of the samples after acid treatment. **a** Left: Im-COF-1. Right: Azo-COF-1. **b** Left: Im-COF-2. Right: Azo-COF-2.

To further examine completeness of the conversion reaction, the COFs were hydrolyzed (Supplementary Section 11). As for Im-COF-1, the solid suspension in DMSO-*d*_6_ disappeared to form a clear and transparent solution upon the addition of deuterium chloride (D_2_O solution), indicating a complete hydrolysis. The peaks corresponding to protons of TPA (10.06 and 8.05 ppm) and 4,4′,4″,4‴-(ethene-1,1,2,2-tetrayl)tetraaniline (ETTA) (7.20 and 7.10 ppm) were identified after the acidic treatment (Fig. 4a, top). The molar ratio of ETTA to TPA was calculated to be 0.5 based on integrals of the peaks, in full agreement with the COF structure. In sharp contrast, Azo-COF-1 remained as a non-hydrolyzable solid after the same treatment, suggesting its much higher chemical stability endowed by the irreversible azo bond (Fig. 4a, bottom). Moreover, no signals corresponding to TPA were observed in the ^1^H NMR spectrum of Azo-COF-1, indicating a complete imine to azo conversion. A similar result was also found for Im-COF-2 and Azo-COF-2, except that no homogeneous solution was obtained after the hydrolysis of Im-COF-2. It is ascribed to the poor solubility of 4,4′,4″,4‴-(pyrene-1,3,6,8-tetrayl)tetraaniline (PTTA) in the solvent. As a result, its signals are very weak (7.80 and 7.63 ppm) (Fig. 4b). The increased chemical stability of the resultant crystallites further corroborates the successful structural conversion from the Im-COFs to the Azo-COFs.

### Photophysical and photocatalysis studies

**Fig. 5:**
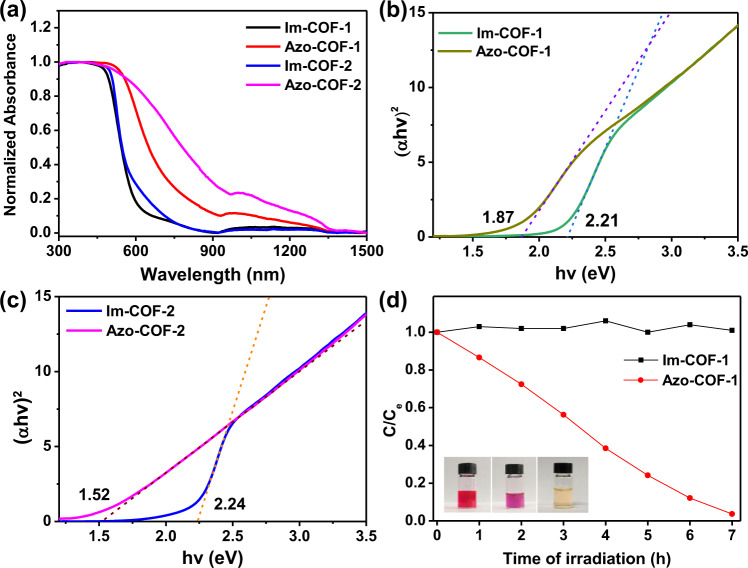
Photophysical and photocatalysis properties. **a** Solid-state UV–vis–NIR DRS. **b**, **c** Tauc plot analysis of the COFs. **d** Comparison of the performance of Im-COF-1 and Azo-COF-1 on photocatalytic degradation of Rh B. Inset: photograph of Rh B solutions of pristine (left), and after photodegradation catalyzed by Im-COF-1 (middle) and Azo-COF-1 (right).

Azopolymers have been revealed to possess narrow bandgap endowed by azo bond^30^. To check such property in Azo-COFs, UV–vis–NIR diffuse reflectance spectra (DRS) were collected for the COFs and model compound **2** (Fig. 5a and Supplementary Section 12), from which we found that the Azo-COFs showed much wider absorption than the Im-COFs, with the former ranging from visible light to near infrared region. The optical band gaps were calculated based on Tauc plot analysis, which gave rise to 2.21, 1.87, 2.24, and 1.52 eV for Im-COF-1, Azo-COF-1, Im-COF-2, and Azo-COF-2, respectively (Fig. 5b, c). In addition, in comparison with model compound **2**, the Azo-COFs exhibited red shifts in their absorption spectra, indicative of more extended conjugated structures (Supplementary Fig. 41). The narrower bandgap of the Azo-COFs inspired us to explore their application in photocatalysis. To this end, photodegradation of rhodamine B (Rh B) catalyzed by Azo-COF-1 was investigated as a demonstration (Fig. 5d and Supplementary Section 13). In the presence of Azo-COF-1, the dye was quickly degraded under visible light irradiation, as revealed by the hypochromatic shift of the peak in the UV–vis spectra (Supplementary Figs. 42, 43). For comparison, photocatalytic performance of Im-COF-1 was also examined. In sharp contrast, it exhibited almost no photocatalytic activity under the similar experimental condition (Fig. 5d and Supplementary Figs. 44, 45). These results clearly demonstrate that azo-linked COFs possess distinct properties from imine-linked COFs and the necessity of the construction of azo-linked COFs.

### Investigation of photocatalytic mechanism

**Fig. 6:**
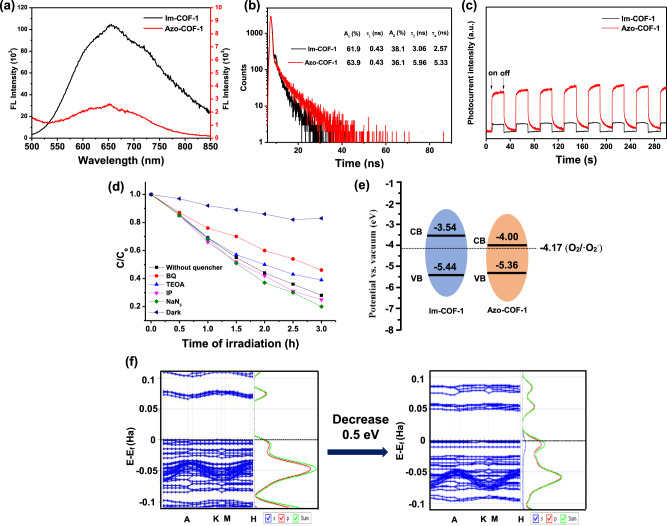
Characterization for the photocatalytic mechanism study. Comparisons of (**a**) steady-state PL spectra excited at 375 nm, (**b**) time-resolved transient PL decay curves, and (**c**) transient photocurrent response measurements between Im-COF-1 and Azo-COF-1. **d** Photocatalytic degradation of RhB by Azo-COF-1 in the presence of different scavengers. **e**, **f** DFT calculations of band structures of Im-COF-1 and Azo-COF-1.

To rationalize the dramatic difference in the photocatalytic behavior of Im-COF-1 and Azo-COF-1, a series of investigations were conducted. As revealed by the photoluminescence (PL) spectra, the emission of Azo-COF-1 was significantly quenched in comparison with that of Im-COF-1, which preliminarily confirmed an efficient suppression of the electron–hole recombination in Azo-COF-1 (Fig. 6a). To gain more information about the charge separation behavior of Im-COF-1 and Azo-COF-1, time-resolved PL decay curves were collected, which indicated that the fluorescence lifetime increased from 2.57 ns of Im-COF-1 to 5.33 ns of Azo-COF-1 (Fig. 6b). The longer lifetime of Azo-COF-1 suggests that it has better performance to suppress the recombination of electron–hole pairs on the excited state than Im-COF-1^50^. This proposal was further supported by transient photocurrent response measurements, where a higher photocurrent of Azo-COF-1 manifests its promoted generation and transfer of photogenerated electron–hole pairs (Fig. 6c). Furthermore, the distinct photocatalytic performance of Azo-COF-1 and Im-COF-1 could also be interpreted by their different energy level structures. On the basis of reactive species capturing experiments, superoxide radical (^**·**^O_2_^−^) was identified as the primary active species in the photocatalytic process (Fig. 6d, Supplementary Fig. 46). Since the ^**·**^O_2_^−^ is formed by the reaction of photoinduced electrons from conductive band (CB) with dissolved O_2_, it could be anticipated that the CB level of Azo-COF-1 matched better with E^0^ (O_2_/^**·**^O_2_^−^ = −4.17 eV vs. Vacuum) than that of Im-COF-1. This assumption was verified by the density functional theory (DFT) calculations, which indicated that Azo-COF-1 had a lower CB level than Im-COF-1 and thus favored the charge transfer process (Fig. 6e). Moreover, the calculations also indicated that the bandgap of Im-COF-1 decreased by 0.5 eV after the transformation, which is close to the experimental result (Fig. 6f). To further corroborate the argumentation, the experiment of photodegradation of RhB catalyzed by Im-COF-1 was re-conducted in the presence of ethylenediaminetetraacetic acid disodium salt (EDTA·2Na, a scavenger of holes). After the addition of EDTA·2Na, a conspicuously arising of degradation of RhB was observed, suggesting that the poor photocatalytic performance of Im-COF-1 alone could be ascribed to the preferential recombination of photoinduced electron–hole pairs (Supplementary Figs. 47, 48).

## Discussion

In summary, an approach to explore new linkage chemistry for COFs have been developed via linker exchange. Based on this approach, a synthetic method for azo-linked COFs has been realized, which leads to a complete transformation from reversible –C=N– to irreversible –N=N– in the COFs. The template effect of precursor COFs provides guidance for the construction of target COFs, through which the crystallization problem could be overcome. Benefiting from the unique property of azo linkage, the Azo-COFs exhibit distinct properties from their corresponding imine-linked counterparts. The azo-linked COFs represent a type of crystalline π-conjugated polymers, which not only enrich the linkage chemistry of COFs, but also provide a platform to explore their application as a class of semiconductors. Moreover, this work provides a useful tool to explore new types of COF linkages inaccessible by the traditional direct polycondensation of monomers, through which development of more novel COF linkages could be expected.

## Methods

### Synthesis of Im-COF-1

The monomers 4,4′,4″,4‴-(ethene-1, 1, 2, 2-tetrayl)tetraaniline (ETTA, 29.5 mg, 0.075 mmol) and terephthalaldehyde (TPA, 20.1 mg, 0.15 mmol) were added into a mixture of dioxane and 6 M HOAc (2/0.2 mL, v/v) in a glass ampoule. After three freeze-pump-thaw cycles, the glass ampoule was sealed under vacuum and allowed to stand at 120 °C for 96 h. After cooling to room temperature, the resulting solid was collected by filtration, followed by immersion in dry dioxane to give Im-COF-1 as a yellow powder after filtration (30.1 mg, 68%). ^13^C-labeled Im-COF-1 was synthesized according to the same procedure using ^3^C-labeled TPA.

### Synthesis of Im-COF-2

4,4′,4″,4‴-(pyrene-1, 3, 6, 8-tetrayl)tetraaniline (PTTA, 22.6 mg, 0.04 mmol), terephthalaldehyde (TPA, 10.7 mg, 0.08 mmol), and a mixture of dioxane and mesitylene (0.5/0.5 mL, v/v) were added into a glass ampoule in sequence. Upon being sonicated for 10 min, aqueous acetic acid (6 M, 0.1 mL) was added as a catalyst. After three freeze-pump-thaw cycles, the glass ampoule was sealed under vacuum and heated at 120 °C for 72 h to afford Im-COF-2 as a yellow powder (30 mg, 98%). The purification process was similar to that of Im-COF-1. ^13^C-labeled Im-COF-2 was synthesized according to the same procedure using ^3^C-labeled TPA.

### Synthesis of Azo-COF-1

Im-COF-1 (10 mg) and DNB (13.8 mg, 3 equiv. of the amount of C=N) were added into a mixture of dioxane/H_2_O/glacial acetic acid (0.8/0.2/0.05 mL, v/v) in a glass ampoule, followed by standing at 120 °C for 3 days. After cooling to ambient temperature, the mixture was filtered, and washed with aqueous NaOH solution (1 M), water, acetone, DMF, and THF in sequence. The as-obtained solid was subject to Soxhlet extraction using CHCl_3_ and THF, each for 12 h, respectively, from which Azo-COF-1 was obtained as a red powder (11.6 mg, theoretical yield: 10.1 mg). Note: The excessive yield is attributed to adsorbed guest molecules (such as the monomer DNB, water and acid molecules) which are hard to remove due to low solubility of DNB and strong binding ability of –N=N– units to water and acid derived from their Lewis base property. The adsorbed DNB was evidenced by XPS. An incomplete conversion was observed when the equivalence of DNB was less than 3 equiv.

### Synthesis of Azo-COF-2

Im-COF-2 (10 mg) and DNB (13.2 mg, 4 equiv. of the amount of C=N units in Im-COF-2) were added into a mixture of dioxane/H_2_O/trifluoroacetic acid (0.8/0.2/0.025 mL, v/v) in a glass ampoule. The following procedure was similar to that described for the synthesis of Azo-COF-1. The Azo-COF-2 was obtained as a brown powder (11.7 mg, theoretical yield: 10.1 mg).

Note: The excessive yield is attributed to adsorbed guest molecules (such as the monomer DNB, water and acid molecules) which are hard to remove due to low solubility of DNB and strong binding ability of –N=N– units to water and acid derived from their Lewis base property. The adsorbed DNB was evidenced by XPS. An incomplete conversion was observed when the equivalence of DNB was less than 4 equiv.

### Hydrolysis experiments

The hydrolysis experiments were performed under acidic condition following the procedure below. To a vial charged with a mixture of the COF sample (10 mg), DMSO-*d*_6_ (1 mL), and DCl (0.1 mL, 12 M in D_2_O) was added. The mixture was stirred at 60 °C for 6 h and the resulting mixture was subjected to ^1^H NMR analysis.

### Photocatalytic degradation experiments

In a typical photocatalytic procedure, the photocatalyst (Azo-COF-1, 10 mg) was dispersed in an aqueous solution of Rh B (25 ppm, 100 mL), followed by magnetically stirring in dark. After reaching adsorption-desorption equilibrium, the system was exposed to a 300 W xenon lamp with an optical cutoff filter (λ ≥ 420 nm). For the collection of UV–vis spectra, 1 mL of aliquot was extracted and centrifuged to remove the photocatalyst at the given intervals. After that, the resulting supernatant was diluted with distilled water (2.0 mL), and then analyzed with a UV–vis spectrophotometer. For comparison, the photocatalytic activity of Im-COF-1 was also assessed following the similar experimental procedure. The performance of photocatalytic degradation of Rh B catalyzed by the COFs was evaluated by plot of C/C_e_ versus time, where C_e_ and C are the concentrations of Rh B at the adsorption–desorption equilibrium and t minutes of the photocatalytic reaction, respectively.

### The photoelectrochemical test

Photocurrent response measurement was performed in a three-electrode electrochemical quartz glass cell. Prior to the test, the photocatalysts (5 mg) were dispersed in 5% nafion (50 uL) and EtOH (0.45 mL) to afford a suspension, which was then dip-coated on the ITO surface and allowed to dry for 24 h at room temperature. Photocurrent measurements were conducted on a CHI660D electrochemical workstation in a standard three-electrode system with the photocatalyst composites as the working electrode, Pt foil as the counter electrode, saturated calomel electrode as the reference electrode, and 0.1 M Na_2_SO_4_ aqueous solution as the electrolyte. Xenon light was used as the light source. The bias voltage was set as 0.2 V.

### The time-resolved photoluminescence (TRPL) decay experiment

The TRPL decay curves were collected on an Edinburgh FLS1000 spectrophotometer with EPL 375 nm as the source. The TRPL decay curves could be described as the Eq. 1 and the average fluorescent lifetime was calculated according to Eq. 2 below:

1 $${{I}}({{{{{\rm{t}}}}}})\,=\,{A}_{1}\exp \left(-\frac{{{{{{\rm{t}}}}}}}{{\tau }_{1}}\right)\,+\,{A}_{2}\exp \left(-\frac{{{{{{\rm{t}}}}}}}{{\tau }_{2}}\right)$$

2 $${\tau }_{a}\,=\,\frac{{A}_{1}{\tau }_{1}^{2}\,+\,{A}_{2}{\tau }_{2}^{2}}{{A}_{1}{\tau }_{1}\,+\,{A}_{2}{\tau }_{2}}$$

### Computational methods

The HOMO, LUMO, and density of states were calculated by spin-polarized DFT method using the Dmol^3^ package. The generalized gradient approximation with Perdew–Burke–Ernzerhof was applied for exchange-correlation energy. DFT semi-core pseudopotential core treatment was implemented for relativistic effects, with C and O calculated in the all-electron method. The double numerical plus polarization (DNP) basis set was used with a “fine” orbital cutoff. Energy, Force, and displacement convergence were 10^−5^ Ha, 0.002 Ha Å^−1^ and 0.005 Å, respectively. All self-consistent field calculations were performed with convergence criterion of 1 × 10^−6^ Ha with smearing.

## Supplementary information

Supplementary Information

## Data Availability

All data supporting the findings of this study are available within the article, as well as the Supplementary Information file, or available from the corresponding authors on request.

## References

[1] Lyle SJ, Waller PJ, Yaghi OM (2019). Covalent organic frameworks: organic chemistry extended into two and three dimensions. Trends Chem..

[2] Ding SY, Wang W (2013). Covalent organic frameworks (COFs): from design to applications. Chem. Soc. Rev..

[3] Kandambeth S, Dey K, Banerjee R (2019). Covalent organic frameworks: chemistry beyond the structure. J. Am. Chem. Soc..

[4] Geng K (2020). Covalent organic frameworks: design, synthesis, and functions. Chem. Rev..

[5] Song Y, Sun Q, Aguila B, Ma S (2019). Opportunities of covalent organic frameworks for advanced applications. Adv. Sci..

[6] Lohse MS, Bein T (2018). Covalent organic frameworks: structures, synthesis, and applications. Adv. Funct. Mater..

[7] Côté AP (2005). Porous, crystalline, covalent organic frameworks. Science.

[8] Du Y (2016). Ionic covalent organic frameworks with spiroborate linkage. Angew. Chem. Int. Ed..

[9] Uribe-Romo FJ (2009). A crystalline imine-linked 3-D porous covalent organic framework. J. Am. Chem. Soc..

[10] Uribe-Romo FJ (2011). Crystalline covalent organic frameworks with hydrazone linkages. J. Am. Chem. Soc..

[11] Jackson KT, Reich TE, El-Kaderi HM (2012). Targeted synthesis of a porous borazine-linked covalent organic framework. Chem. Commun..

[12] Zhuang X (2016). A two-dimensional conjugated polymer framework with fully sp^2^-bonded carbon skeleton. Polym. Chem..

[13] Jin E (2017). Two-dimensional sp^2^ carbon–conjugated covalent organic frameworks. Science.

[14] Liu H (2018). Covalent organic frameworks linked by amine bonding for concerted electrochemical reduction of CO_2_. Chem.

[15] Jiang S-Y (2019). Aminal-linked covalent organic frameworks through condensation of secondary amine with aldehyde. J. Am. Chem. Soc..

[16] Rao MR, Fang Y, Feyter SD, Perepichka DF (2017). Conjugated covalent organic frameworks via michael addition–elimination. J. Am. Chem. Soc..

[17] Waller PJ (2016). Chemical conversion of linkages in covalent organic frameworks. J. Am. Chem. Soc..

[18] Nagai A (2013). A squaraine-linked mesoporous covalent organic framework. Angew. Chem. Int. Ed..

[19] Fang Q (2014). Designed synthesis of large-pore crystalline polyimide covalent organic frameworks. Nat. Commun..

[20] Li X-T (2020). Construction of covalent organic frameworks via three-component one-pot strecker and povarov reactions. J. Am. Chem. Soc..

[21] Zhang B (2018). Crystalline dioxin-linked covalent organic frameworks from irreversible reactions. J. Am. Chem. Soc..

[22] Guan X (2019). Chemically stable polyarylether-based covalent organic frameworks. Nat. Chem..

[23] Zhao C, Lyu H, Ji Z, Zhu C, Yaghi OM (2020). Ester-linked crystalline covalent organic frameworks. J. Am. Chem. Soc..

[24] Roeser J (2017). Anionic silicate organic frameworks constructed from hexacoordinate silicon centres. Nat. Chem..

[25] Zhou D (2019). Synthesis of C–C bonded two-dimensional conjugated covalent organic framework films by Suzuki polymerization on a liquid-liquid. Interface Angew. Chem. Int. Ed..

[26] Yuan C (2021). Crystalline C–C and C=C bond-linked chiral covalent organic frameworks. J. Am. Chem. Soc..

[27] Yamada M (2008). Photomobile polymer materials: towards light-driven plastic motors. Angew. Chem. Int. Ed..

[28] Wang D, Wang X (2013). Amphiphilic azo polymers: molecular engineering, self-assembly and photoresponsive properties. Prog. Polym. Sci..

[29] Das G (2019). Azobenzene-equipped covalent organic framework: light-operated reservoir. J. Am. Chem. Soc..

[30] Fliegl H, Köhn A, Hättig C, Ahlrichs R (2003). Ab initio calculation of the vibrational and electronic spectra of trans- and cis-azobenzene. J. Am. Chem. Soc..

[31] Andjaba JM (2021). Catalytic synthesis of conjugated azopolymers from aromatic diazides. J. Am. Chem. Soc..

[32] Singh V (2021). Thiazole-linked covalent organic framework promoting fast two-electron transfer for lithium-organic batteries. Adv. Energy Mater.

[33] Wan Y (2020). A simple molecular design strategy for two-dimensional covalent organic framework capable of visible-light-driven water splitting. J. Am. Chem. Soc..

[34] Chandra S (2014). Phosphoric acid loaded Azo (–N=N–) based covalent organic framework for proton conduction. J. Am. Chem. Soc..

[35] Kandambeth S (2017). Selective molecular sieving in self-standing porous covalent-organic-framework membranes. Adv. Mater..

[36] Biswal BP (2015). Pore surface engineering in porous, chemically stable covalent organic frameworks for water adsorption. J. Mater. Chem. A.

[37] Ge R (2016). Target synthesis of an Azo (N=N) based covalent organic framework with high CO_2_-over-N_2_ selectivity and benign gas storage capability. J. Chem. Eng. Data.

[38] Yang Y (2020). Combined intrinsic and extrinsic proton conduction in robust covalent organic frameworks for hydrogen fuel cell applications. Angew. Chem. Int. Ed..

[39] Mills C (1895). XCIII.—Some new azo-compounds. J. Chem. Soc..

[40] Yoshiro O, Yasuo T (1958). Kinetics of the condensation of anilines with nitrosobenzenes to form azobenzenes. J. Am. Chem. Soc..

[41] Qian C (2017). Toward covalent organic frameworks bearing three different kinds of pores: the strategy for construction and COF-to-COF transformation via heterogeneous linker exchange. J. Am. Chem. Soc..

[42] Qian H-L, Meng F-L, Yang C-X, Yan X-P (2020). Irreversible amide-linked covalent organic framework for selective and ultrafast gold recovery. Angew. Chem. Int. Ed..

[43] Zhai Y (2019). Construction of covalent-organic frameworks (COFs) from amorphous covalent organic polymers via linkage replacement. Angew. Chem. Int. Ed..

[44] Zhou T-Y (2014). One-step construction of two different kinds of pores in a 2D covalent organic framework. J. Am. Chem. Soc..

[45] Ascherl L (2016). Molecular docking sites designed for the generation of highly crystalline covalent organic frameworks. Nat. Chem..

[46] Auras F (2016). Synchronized offset stacking: a concept for growing large-domain and highly crystalline 2D covalent organic frameworks. J. Am. Chem. Soc..

[47] Ueno K (2002). Polyazobenzenes. III. Infrared absorption spectra of some polyazobenzenes. J. Am. Chem. Soc..

[48] Brant P, Feltham RD (1976). X-ray photoelectron spectra of aryldiazo derivatives of transition metals. J. Organometal. Chem..

[49] Camalli M (1990). Adducts of tin(IV) and organotin(IV) derivatives with 2,2′-azopyridine II. Crystal and molecular structure of SnMe_2_Br_2_AZP and further mössbauer and photoelectronic spectroscopic studies. Inorg. Chim. Acta.

[50] Zhang Z (2017). A nonmetal plasmonic Z-scheme photocatalyst with UV- to NIR-driven photocatalytic protons reduction. Adv. Mater..

[51] Batich CD, Donald DS (1984). X-ray photoelectron spectroscopy of nitroso compounds: relative ionicity of the closed and open forms. J. Am. Chem. Soc..

