# Supplementary material for: Toward azo-linked covalent organic frameworks by developing linkage chemistry via linker exchange
Source: Nat Commun. 2022 Apr 21;13:2180. doi: 10.1038/s41467-022-29814-3 (PMC9023542; doi:10.1038/s41467-022-29814-3)
Supplement: Supplementary file 1 — Supplementary Information [file 41467_2022_29814_MOESM1_ESM.pdf]

Supplementary Information for

Toward azo-linked covalent organic frameworks by developing linkage chemistry via linker exchange

Zhou *et al.*

Zhi-Bei Zhou, Peng-Ju Tian, Jin Yao, Ya Lu, Qiao-Yan Qi, and Xin Zhao*

Key Laboratory of Synthetic and Self-Assembly Chemistry for Organic Functional Molecules, Center for Excellence in Molecular Synthesis, Shanghai Institute of Organic Chemistry, University of Chinese Academy of Sciences, Chinese Academy of Sciences, 345 Lingling Road, Shanghai 200032, China

Table of Content

Supplementary Section 1. Materials and Instruments..3-4

Supplementary Section 2. Attempts to synthesize Azo-COFs via Mills reaction 5-7

Supplementary Section 3. Model reaction 8-10

Supplementary Section 4. Synthesis of ^{13}C-labeled TPA 11-12

Supplementary Section 5. Solid-state ^{13}C CP-MAS NMR results 13

Supplementary Section 6. Characterization with Raman spectroscopy 14-15

Supplementary Section 7. PXRD characterization of Im-COFs and Azo-COFs 16-19

Supplementary Section 8. XPS characterization 20

Supplementary Section 9. N_2 sorption isotherm experiments 21-24

Supplementary Section 10. SEM images...25

Supplementary Section 11. Hydrolysis experiments 26-28

Supplementary Section 12. Solid-state UV-vis-NIR DRS 29

Supplementary Section 13. Photocatalytic degradation study 30-34

Supplementary Section 14. Fractional atomic coordinates of the COFs 35-38

Supplementary References 39

Supplementary Section 1. Materials and Instruments

Materials: All the chemicals commercially available were used directly without further purification. *N*,*N*-dimethylformamide-(carbonyl-^{13}C) (99% ^{13}C) was purchased from Sigma Aldrich. 2-(4-bromophenyl)-1,3-dioxolane and aniline was purchased from TCI. Terephthalaldehyde and n-butyllithium (2.5 M in hexane) was purchased from Innochem. (1E,4E)-benzoquinone dioxime was purchased from Aladdin. Common organic solvents were purchased from Macklin.

Powder X-ray diffraction

Powder X-ray diffraction (PXRD) experiment was performed on a PANalytical X'Pert Powder system using Cu/Kα (λ = 0.1542 nm) radiation with a step size of 0.026°. The samples were spread on the square recess of a holder before the measurements.

Fourier transform infrared spectroscopy

Fourier transform infrared (FT-IR) spectra were collected with a Nicolet iS10 spectrometer. The samples were prepared as KBr pellets.

Solid state ^{13}C CP-MAS nuclear magnetic resonance

Solid state ^{13}C CP-MAS nuclear magnetic resonance (NMR) measurement was carried out on an Agilent DD2 600 Solid NMR System with 4mm zirconia rotors. The spinning rate is 7-10 kHz and the contact time is 3 ms.

Raman spectroscopy

Raman spectroscopy was performed with a ThermoScientific DXR Raman Microscopy instrument with a laser as a source. The Laser wavelength and power varied from the samples.

X-ray photoelectron spectroscopy

X-ray photoelectron spectroscopy (XPS) was conducted on a Thermo Scientific K-Alpha^{+} with the monochromatic Al/Kα source (energy 1486.6 eV) under ultrahigh vacuum (analytical chamber pressure 10^{-9} mbar).

Scanning electron microscopy

Scanning electron microscopy (SEM) was carried out using a XL30 FEG scanning electron microscope. The samples were dispersed over a slice of conductive adhesive adhered to a flat copper platform sample holder and then coated with gold using a sputter 9 coater (ambient temperature, 85 torr pressure in a nitrogen atmosphere, sputtered for 80 s from a solid gold target at a current of 20 mA) before being submitted to SEM characterization.

Nitrogen sorption measurement

Nitrogen adsorption-desorption isotherm measurement was performed on a Quantanchrome autosorb IQ system. The isotherms were collected at 77 K under a liquid nitrogen bath. The specific surface areas were calculated utilizing the Brunauer-Emmett-Teller (BET) method and pore size distribution profiles were derived from the adsorption data using density functional theory (DFT) model.

Solid-state UV-vis-NIR diffuse reflectance spectroscopy

Solid-state UV-vis-NIR diffuse reflectance spectra (DRS) were collected on a Hitachi UH4150 UV–vis-NIR spectrophotometer equipped with integrating sphere using $BaSO_4$ as a reflectance standard.

Liquid UV-vis adsorption spectroscopy

Liquid UV-vis adsorption spectra were collected on a Unico UV-4802 spectrophotometer.

Photoluminescence spectroscopy

Photoluminescence (PL) spectra were collected on an Edinburgh FLS1000 spectrophotometer. Steady-state PL spectra and time-resolved PL decay curves were recorded with the excitation wavelength of 375 nm using EPL source.

Supplementary Section 2. Attempts to synthesize Azo-COFs via Mills reaction

Synthesis of 1,4-dinitrosobenzene (**DNB**):

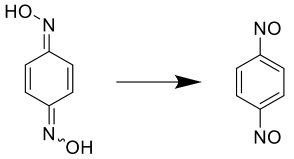

The procedure was conducted according to literature with modifications.[1] A suspension of (1E,4E)-benzoquinone dioxime (5.0 g), H_2O_2 (15 mL, 30%), concentrated HCl (10 mL), and CH_2Cl_2 (50 mL) was stirred at 50 ºC for 4 hours. Then the mixture was filtered to afford a yellow solid (4.3 g, 86.4%). ^{1}H NMR (400 M, $CDCl_3$) δ 8.14 (s, 4H). ^{13}C NMR (100 M, $CDCl_3$) δ 122.35, 149.30. LR-MS (EI): m/z Calcd. for $C_6H_4N_2O_2$: 136. Found: 136. HR-MS (EI): m/z Calcd. 136.0267. Found: 136.0265.

Condensation reactions:

General procedure: A mixture of tetragonal monomer 4,4',4'',4'''-(ethene-1,1,2,2-tetrayl)tetraaniline (**ETTA**, 0.04 mmol) or 4,4',4'',4'''-(pyrene-1,3,6,8-tetrayl)tetraaniline (**PTTA**, 0.03 mmol), **DNB** (2 equiv. of the amount of **ETTA** or **PTTA**), corresponding solvent (1.0 mL) and catalyst (0.1 mL) was added into a 10 mL glass ampoule, followed by ultrasonication for 5 minutes. After three freeze-pump-thaw cycles, the glass ampoule was sealed under vacuum and allowed to stand at corresponding temperature for a period of time. After cooling to room temperature, the resulting solid was collected by filtration, washed with water, acetone, and THF, respectively. Then the solid was dried under vacuum before being subjected to PXRD measurement.

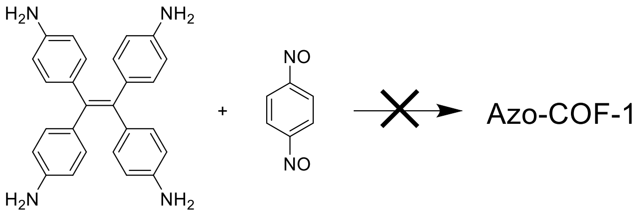

Entry	Solvent	Catalyst	Temperature (℃)	Time (days)	Crystallinity
1	dioxane	6 M HOAc	120	3	amorphous
2	o-dichlorobenzene	6 M HOAc	120	3	amorphous
3	dioxane/ mesitylene (0.5/0.5, v/v)	6 M HOAc	120	3	amorphous
4	o-dichlorobenzene/ n-butyl alcohol (0.5/0.5, v/v)	6 M HOAc	120	3	amorphous
5	toluene	6 M HOAc	120	3	amorphous
6	NMP/ mesitylene (0.5/0.5, v/v)	6 M HOAc	120	3	amorphous
7	o-dichlorobenzene/ mesitylene (0.5/0.5, v/v)	6 M HOAc	120	3	amorphous
8	o-xylene	6 M HOAc	120	3	amorphous
9	dioxane	3 M HOAc	120	3	amorphous
10	dioxane/ mesitylene (0.5/0.5, v/v)	3 M HOAc	120	3	amorphous
11	dioxane/ mesitylene (0.5/0.5, v/v)	9 M HOAc	120	3	amorphous
12	dioxane	6 M HOAc	150	3	amorphous
13	o-dichlorobenzene/ n-butyl alcohol (0.5/0.5, v/v)	6 M HOAc	150	3	amorphous
14	o-dichlorobenzene/ mesitylene (0.5/0.5, v/v)	6 M HOAc	150	3	amorphous
15	dioxane/ mesitylene (0.5/0.5, v/v)	6 M HOAc	120	5	amorphous
16	o-dichlorobenzene/ n-butyl alcohol (0.5/0.5, v/v)	6 M HOAc	120	5	amorphous
17	dioxane/H_2O (0.8/0.2, v/v)	HOAc	120	3	amorphous

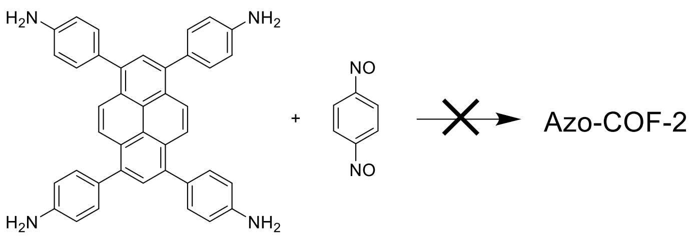

Entry	Solvent	Catalyst	Temperature (℃)	Time (days)	Crystallinity
1	dioxane	6 M HOAc	120	3	amorphous
2	dioxane/mesitylene (0.5/0.5, v/v)	6 M HOAc	120	3	amorphous
3	o-dichlorobenzene/ mesitylene (0.5/0.5, v/v)	6 M HOAc	120	3	amorphous
4	o-dichlorobenzene/ n-butyl alcohol (0.5/0.5, v/v)	6 M HOAc	120	3	amorphous
5	mesitylene	6 M HOAc	120	5	amorphous
6	Toluene	6 M HOAc	120	5	amorphous
7	o-xylene	6 M HOAc	120	5	amorphous
8	NMP/ mesitylene (0.5/0.5, v/v)	6 M HOAc	120	5	No solid
9	dioxane/mesitylene (0.5/0.5, v/v)	HOAc	120	3	amorphous
10	dioxane/H_2O (0.8/0.2, v/v)	CF_3COOH	120	3	amorphous
11	dioxane/mesitylene (0.5/0.5, v/v)	CF_3COOH	120	3	amorphous

Supplementary Section 3. Model reaction

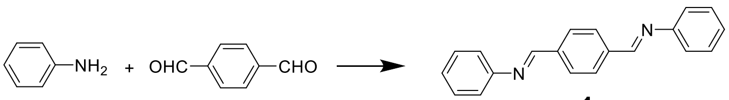

1

(1E,1'E)-1,1'-(1,4-phenylene)bis(N-phenylmethanimine) (**1**): Terephthalaldehyde (2.0 g, 14.9 mmol), $MgSO_4$ (9.0 g, 74.6 mmol), aniline (2.8 mL, 31.3 mmol), and CH_2Cl_2 (30 mL) were added into a 100 mL vial. The mixture was stirred under ambient temperature for 24 hours, followed by filtration under reduced pressure. The crude product obtained by rotary evaporation under vacuum was recrystallized in CH_2Cl_2/hexane to afford **1** as a white solid (1.9 g, 44%). ^{1}H NMR (400 M, DMSO-d_6) δ 8.72 (s, 2H), 8.09 (s, 4H), 7.47-7.43 (m, 4H), 7.33-7.26 (m, 6H). ^{13}C NMR (100 M, DMSO-d_6) δ 160.62, 151.73, 138.96, 129.81, 129.58, 126.88, 121.65. LR-MS (EI): m/z Calcd. for $C_{20}H_{16}N_2$: 284. Found: 284. HR-MS (EI): m/z Calcd. for $C_{20}H_{16}N_2$: 284.1308. Found: 284.1311.

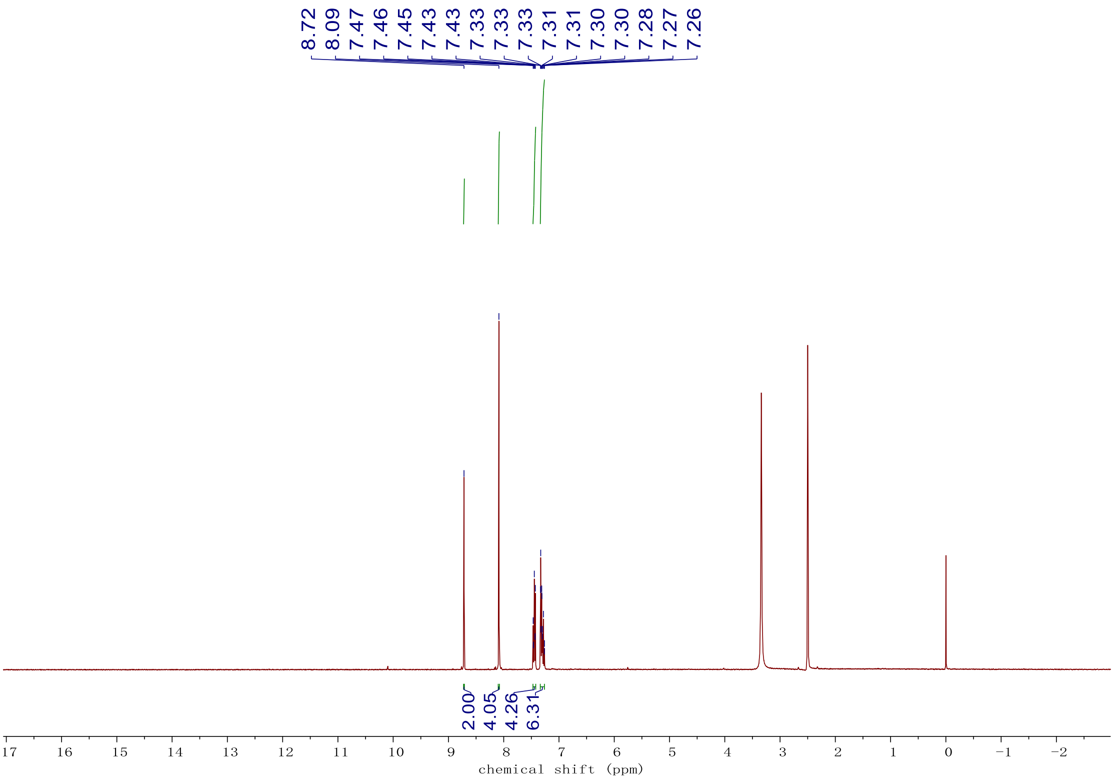

Supplementary Figure 1. ^{1}H NMR spectrum (400 MHz, DMSO-d_6) of compound **1**.

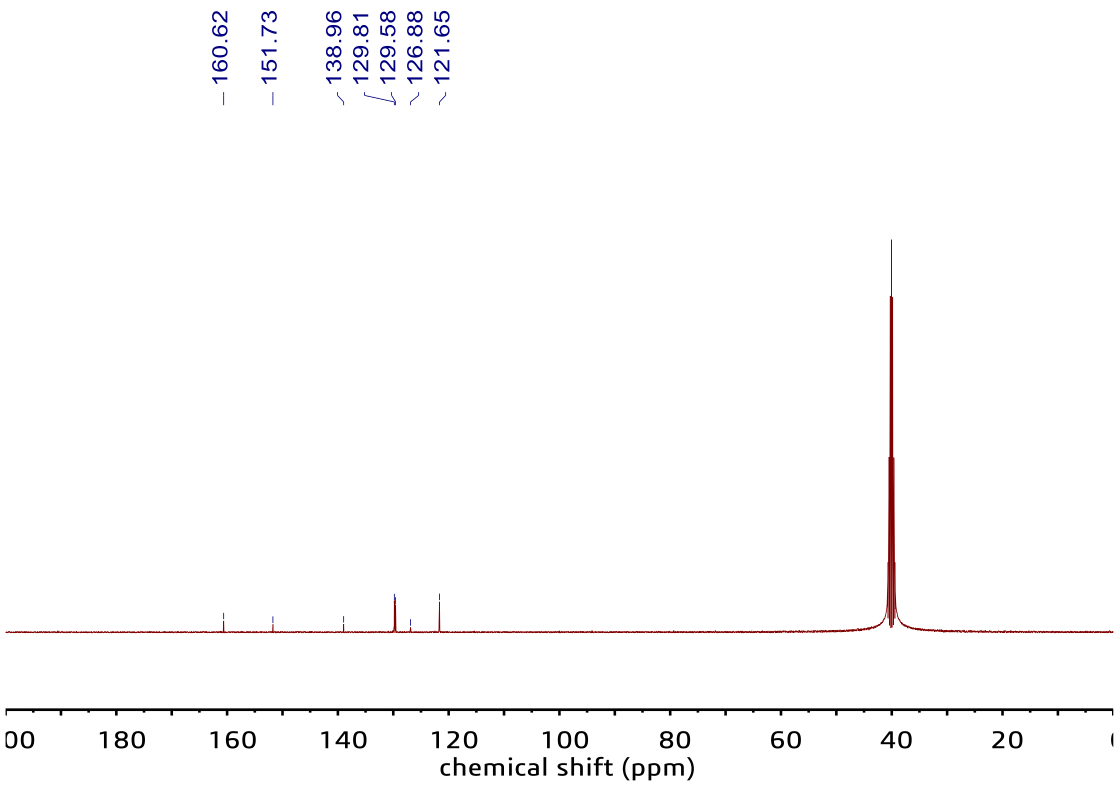

Supplementary Figure 2. ^{13}C NMR spectrum (100 MHz, DMSO-d_6) of compound **1**.

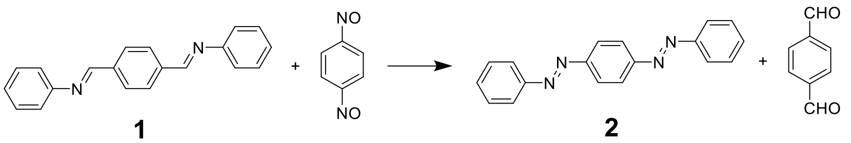

1,4-bis((E)-phenyldiazenyl)benzene (**2**): To a solution of compound **1** (0.5 g, 1.7 mmol), **DNB** (0.7 g, 5.3 mmol) in EtOH (40 mL) and water (2 mL), glacial acetic acid (2 mL) were added. The mixture was heated at 80 ºC under inert atmosphere for 2 days. After completion of the reaction, the solvent was removed under rotary evaporation and the resultant mixture was purified by flash column chromatography (petroleum ether/ethyl acetate = 40/1) to give compound **2** as a reddish solid (0.3 g, 56%). ^1H NMR (400 M, DMSO-d_6) δ 8.12 (s, 4H), 7.98-7.95 (m, 4H), 7.66-7.62 (m, 6H). ^{13}C NMR (100 M, DMSO-d_6) δ 153.79, 152.80, 131.51, 129.26, 123.86, 123.15. LR-MS (EI): m/z Calcd. for $C_{18}H_{14}N_4$: 286. Found: 286. HR-MS (EI): m/z Calcd. for $C_{18}H_{14}N_4$: 286.1213. Found: 286.1207. Note: the reaction condition for the conversion was not optimized.

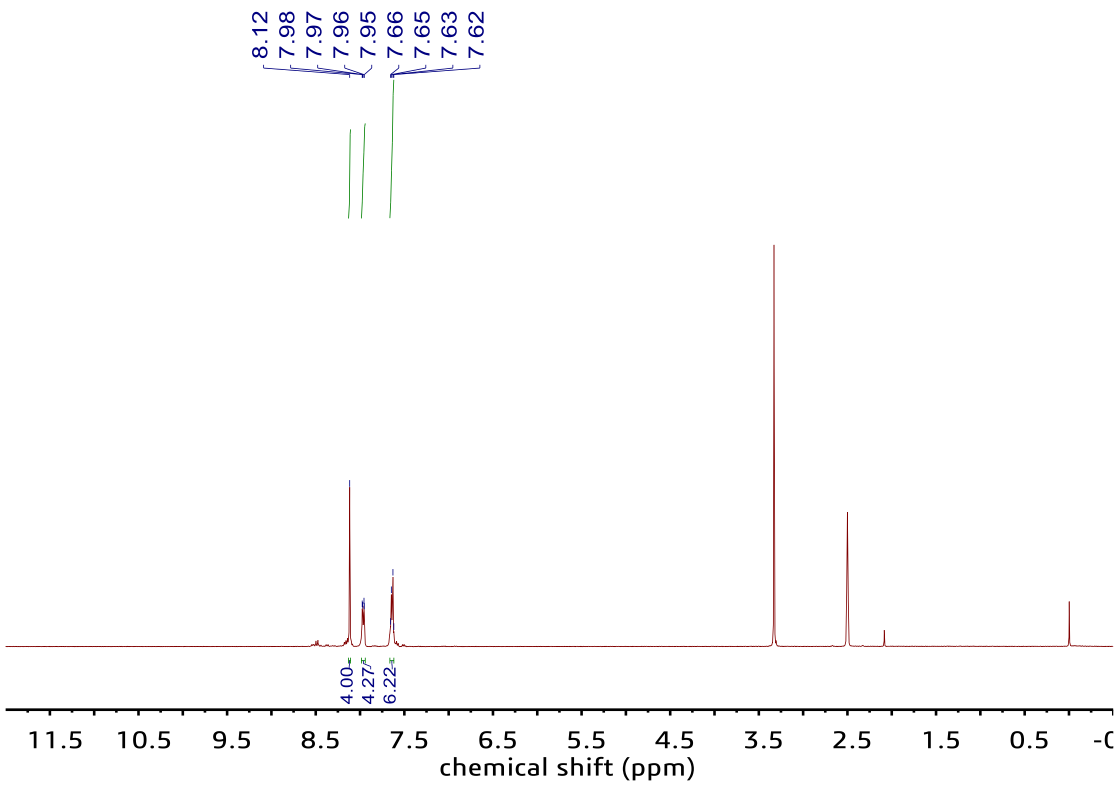

Supplementary Figure 3. ^{1}H NMR spectrum (400 MHz, DMSO-d_6) of compound **2**.

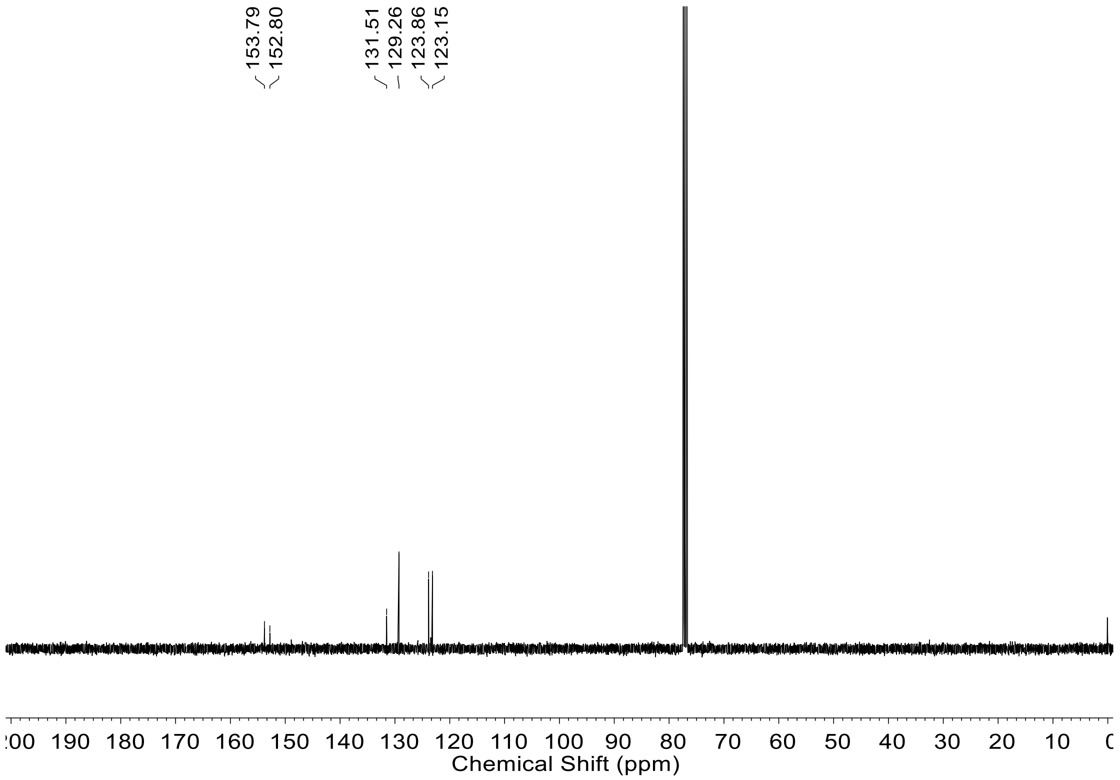

Supplementary Figure 4. ^{13}C NMR spectrum (100 MHz, DMSO-d_6) of compound **2**.

Supplementary Section 4. Synthesis of ^{13}C-labeled TPA[2]:

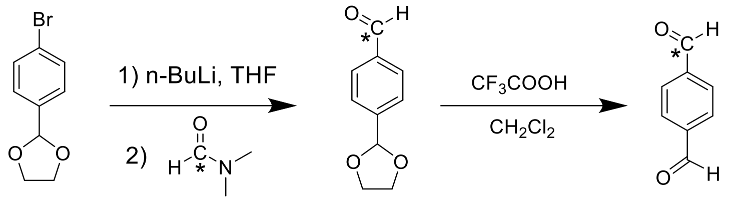

A solution of n-butyllithium in hexane (2.5 M, 2.1 mL, 5.24 mmol) was added dropwise into a mixture of 2-(4-bromophenyl)-1,3-dioxolane (1.0 g, 4.37 mmol) in 50 mL dry THF at -78 °C. After stirring for one hour at -78 °C, ^{13}C-labeled N,N-dimethylformamide (0.53 mL,6.75 mmol) was added. The suspension was allowed to warm to room temperature. After stirring for two hours, the reaction was quenched by slowly adding 20 mL water. The resultant mixture was then subject to rotation evaporation to remove THF, followed by extraction with CH_2Cl_2 (3×50 mL). The combined organic layers were dried over Na_2SO_4 and the evaporation of solvent gave a yellow oil. The as-prepared oil was then added into a mixture of CH_2Cl_2 (10 mL) and CF_3COOH (7 mL). The brown mixture was stirring at ambient temperature for 5 hours. Then water and CH_2Cl_2 were added to separate the organic phase, dried over Na_2SO_4 and evaporated to give crude product, which was purified by column chromatography (CH_2Cl_2 as eluent). Yield: 405 mg, 69%. ^{1}H NMR (400 M, $CDCl_3$) δ 10.14 (s, 1H), 10.14 (d, *J* = 176 Hz, 1H), 8.05 (d, *J* = 4 Hz, 4H). ^{13}C NMR (100 M, $CDCl_3$) δ 191.55, 140.11, 130.24. LR-MS (EI): m/z Calcd. for $C_7{}^{13}CH_6O_2$ 135. Found. 135. HR-MS (EI): m/z Calcd. 135.0396. Found. 135.0397

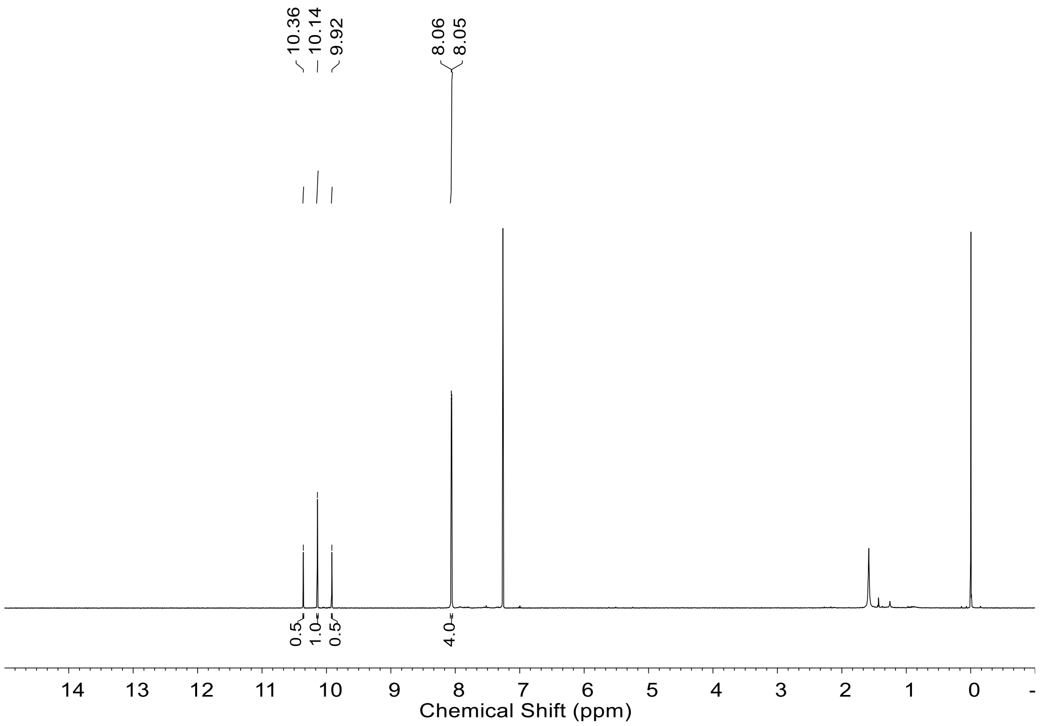

Supplementary Figure 5. ^{1}H NMR spectrum (400 MHz, $CDCl_3$) of ^{13}C-labeled **TPA**.

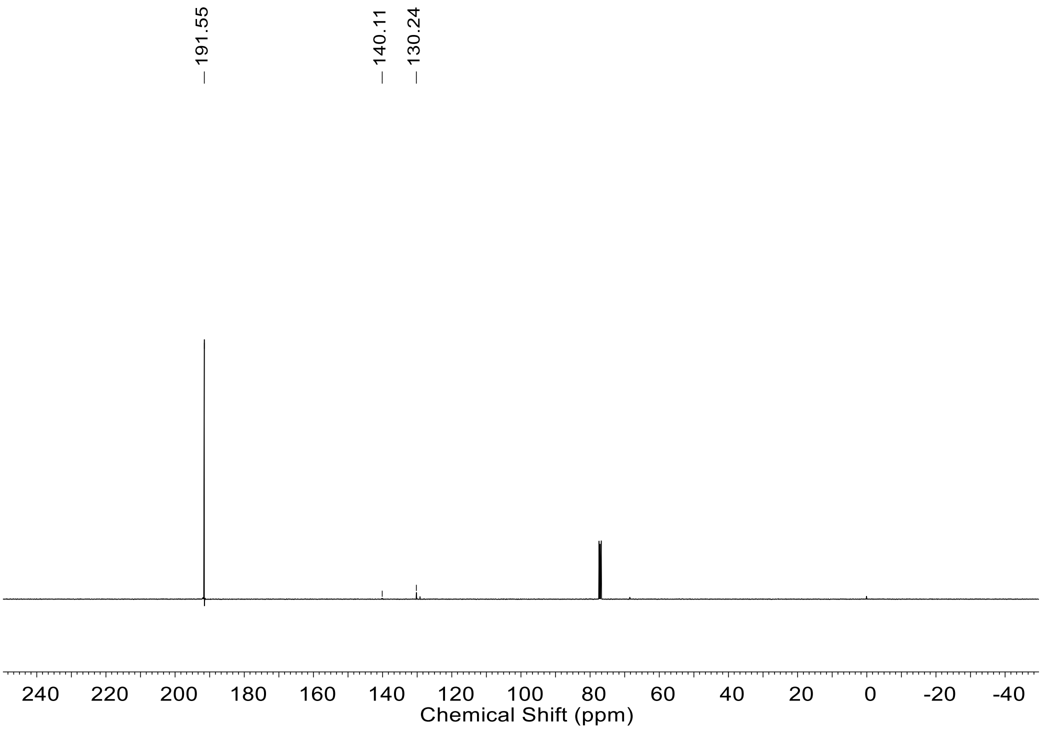

Supplementary Figure 6. ^{13}C NMR spectrum (100 MHz, $CDCl_3$) of ^{13}C-labeled **TPA**.

Supplementary Section 5. Solid-state ^{13}C CP-MAS NMR results

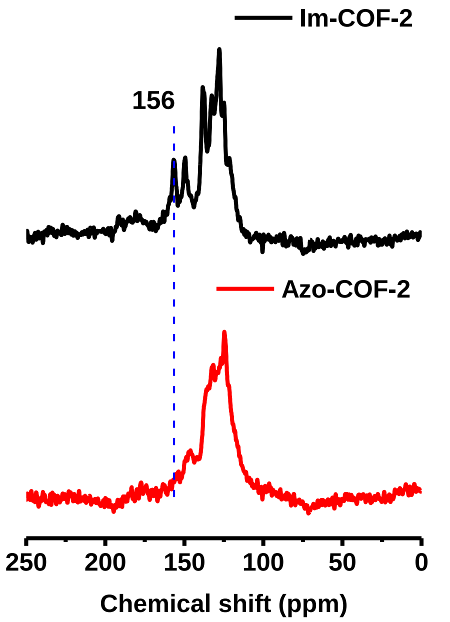

Supplementary Figure 7. Comparison of ^{13}C CP-MAS NMR spectra between **Im-COF-2** and **Azo-COF-2** (natural abundance).

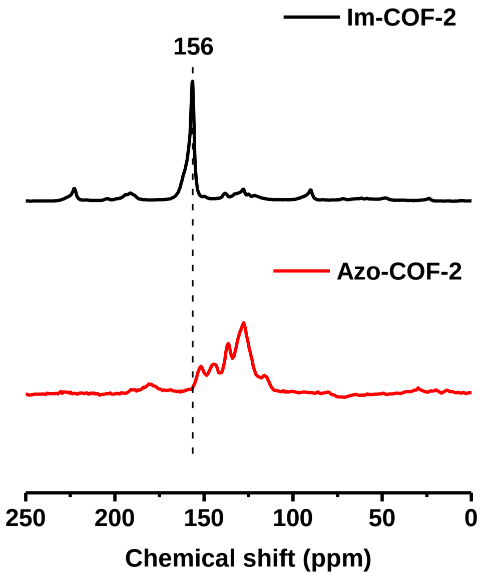

Supplementary Figure 8. Comparison of ^{13}C CP-MAS NMR spectra between **Im-COF-2** and **Azo-COF-2** (half of the imine carbon atoms were labeled with ^{13}C).

Supplementary Section 6. Characterization with Raman spectroscopy

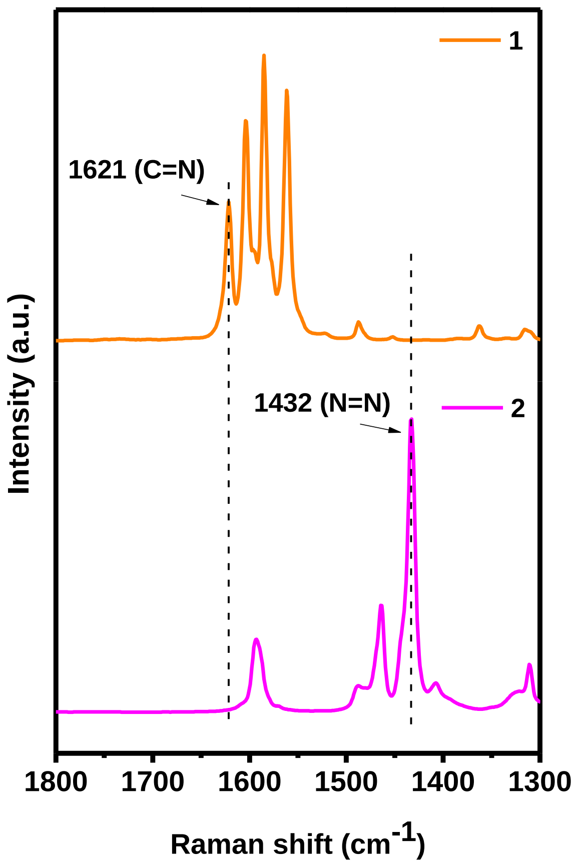

Supplementary Figure 9. Comparison of Raman spectra between compounds **1** and **2** of the model reaction.

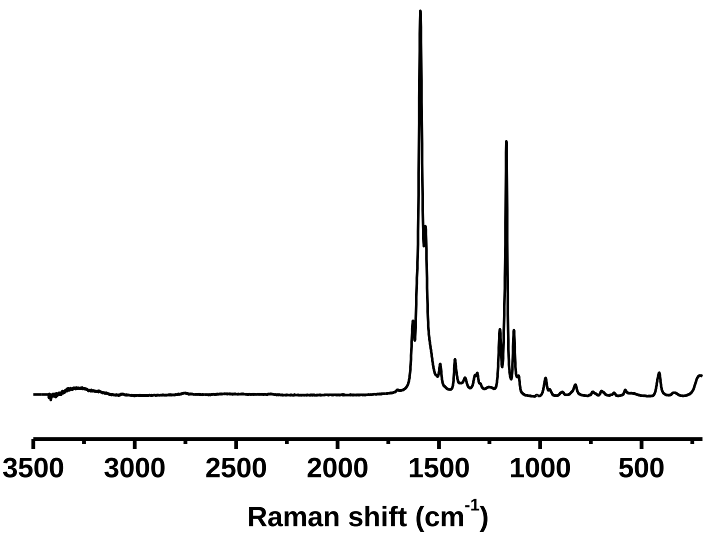

Supplementary Figure 10. Full Raman spectrum of **Im-COF-1** (780 nm laser with a power of 20.0 mw).

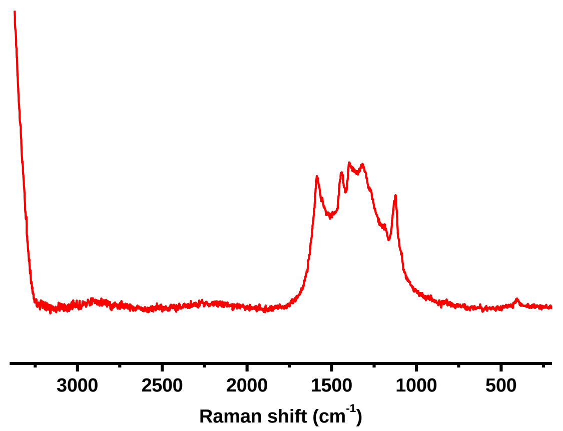

Supplementary Figure 11. Full Raman spectrum of **Azo-COF-1** (633 nm laser with a power of 5.0 mw).

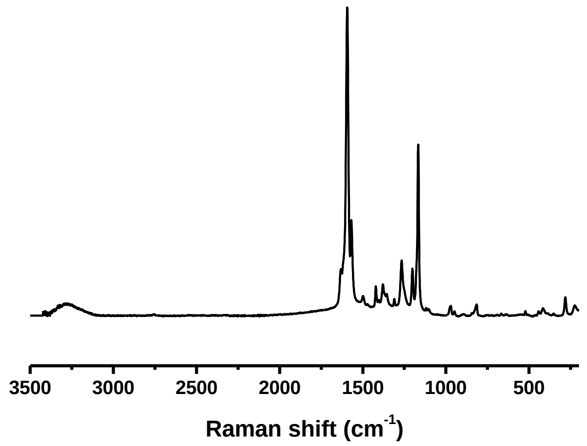

Supplementary Figure 12. Full Raman spectrum of **Im-COF-2** (780 nm laser with a power of 20.0 mw).

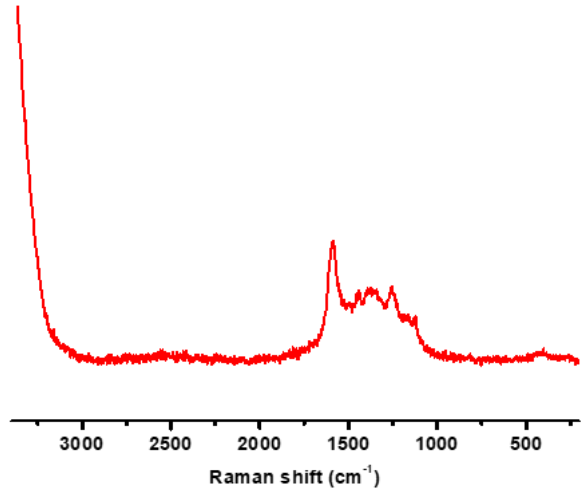

Supplementary Figure 13. Full Raman spectrum of **Azo-COF-2** (532 nm laser with a power of 5.0 mw).

Supplementary Section 7. PXRD characterization of Im-COFs and Azo-COFs

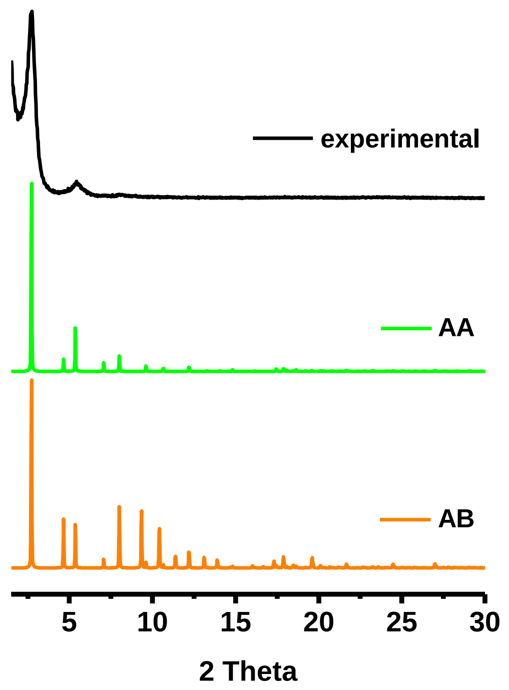

Supplementary Figure 14. Experimental and simulated PXRD patterns of **Im-COF-1**.

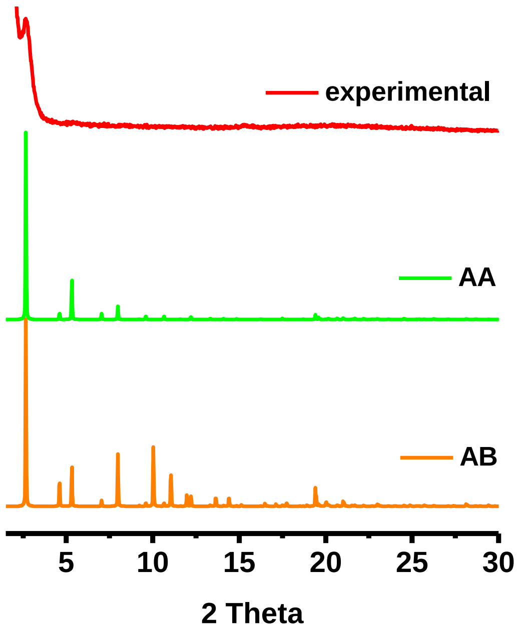

Supplementary Figure 15. Experimental and simulated PXRD patterns of **Azo-COF-1**.

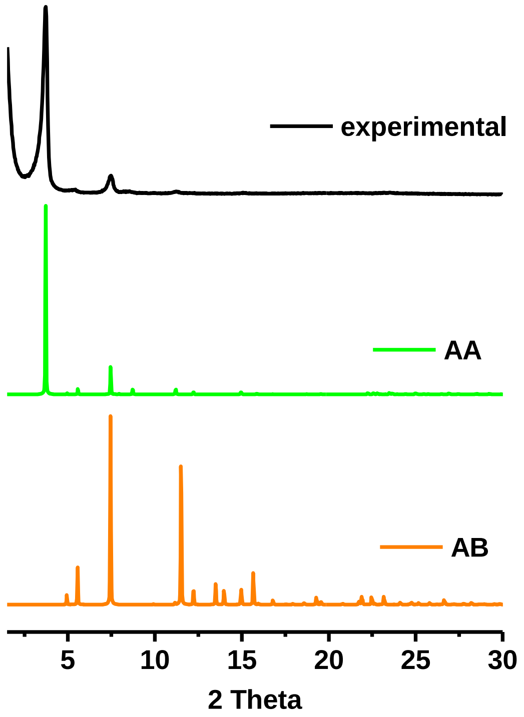

Supplementary Figure 16. Experimental and simulated PXRD patterns of **Im-COF-2**.

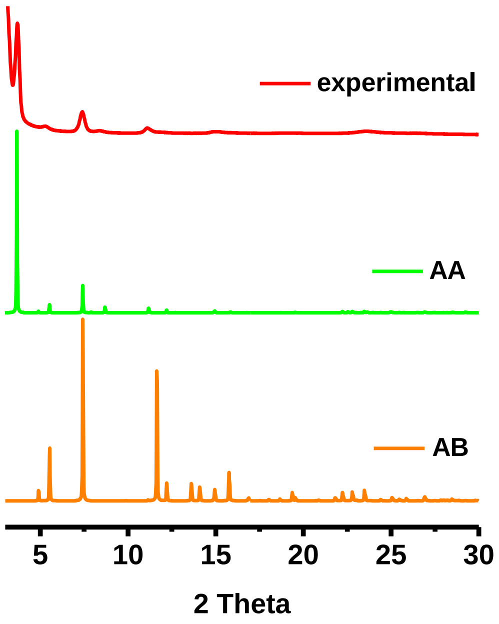

Supplementary Figure 17. Experimental and simulated PXRD patterns of **Azo-COF-2**.

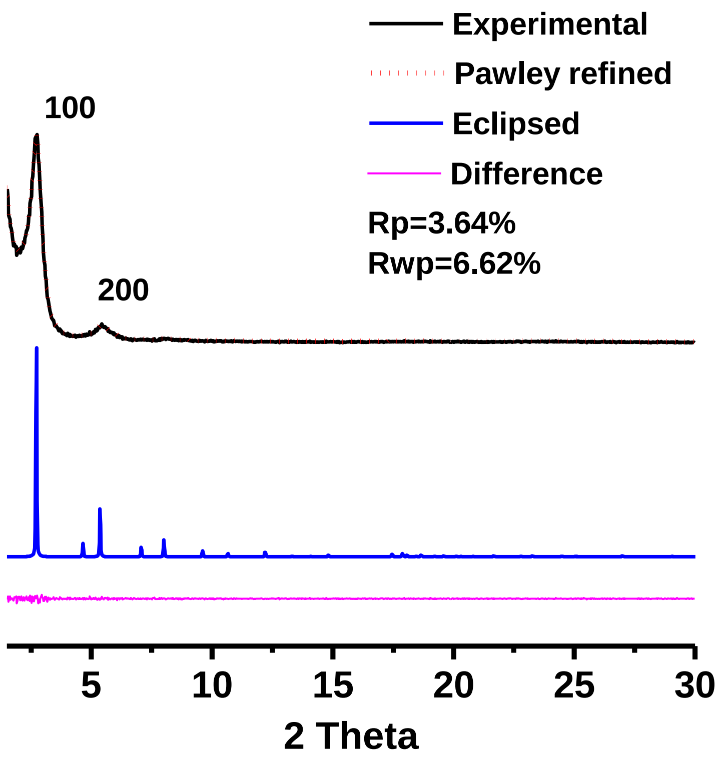

Supplementary Figure 18. Experimental and refined PXRD patterns of **Im-COF-1**.

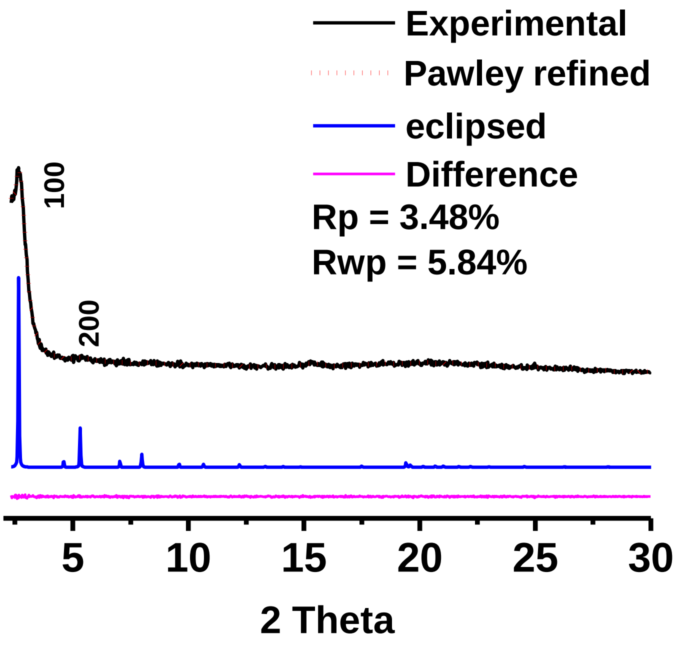

Supplementary Figure 19. Experimental and refined PXRD patterns of **Azo-COF-1**.

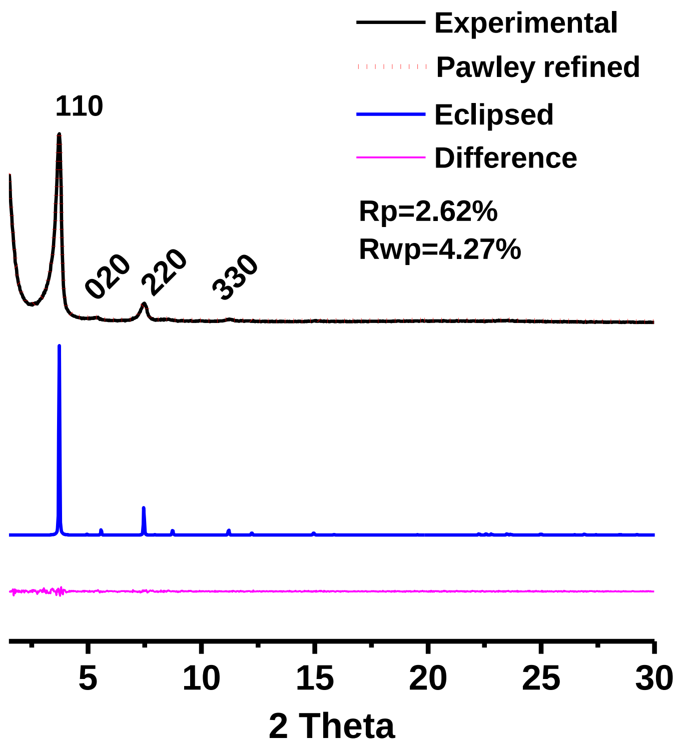

Supplementary Figure 20. Experimental and refined PXRD patterns of **Im-COF-2**.

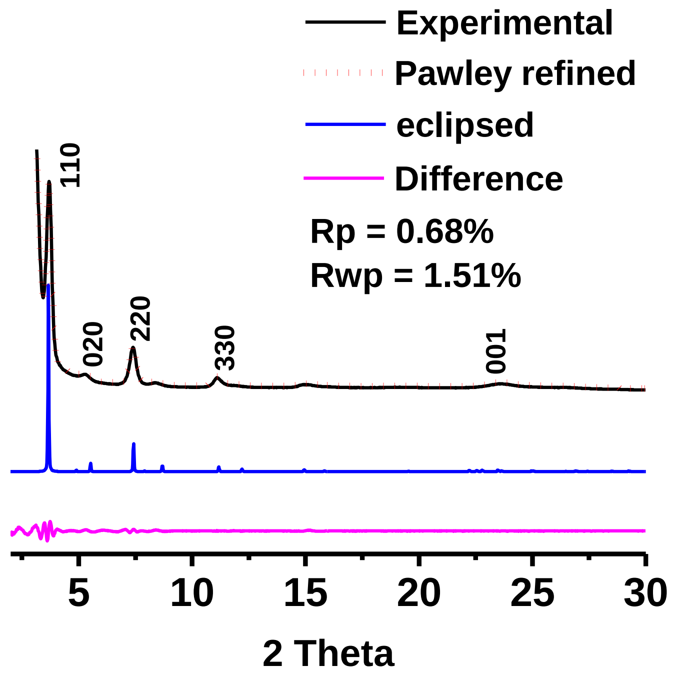

Supplementary Figure 21. Experimental and refined PXRD patterns of **Azo-COF-2**.

Supplementary Section 8. XPS characterization

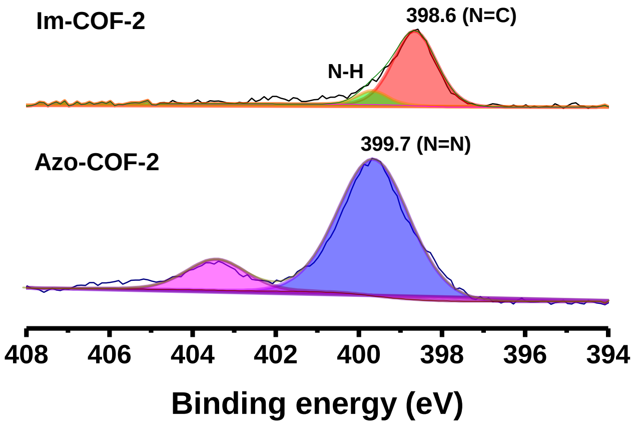

Supplementary Figure 22. Comparison of high resolution XPS spectra of N1*s* between **Im-COF-2** and **Azo-COF-2**.

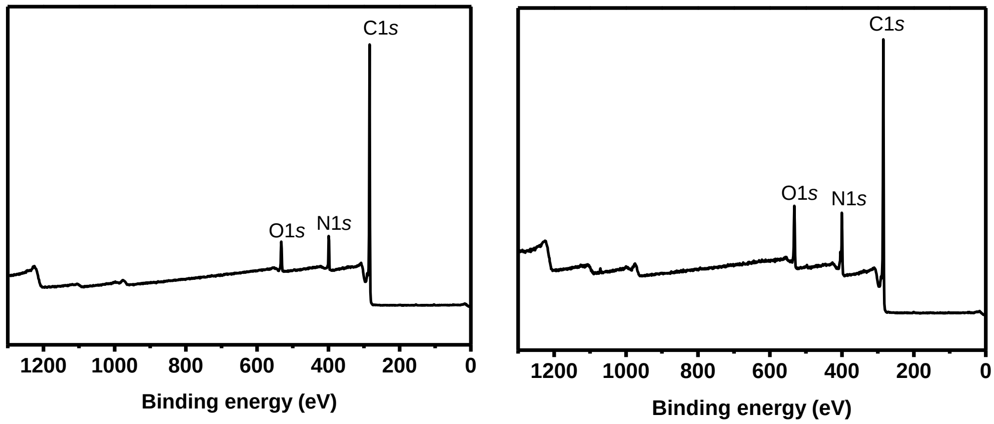

Supplementary Figure 23. XPS survey of **Im-COF-1** (left) and **Azo-COF-1** (right).

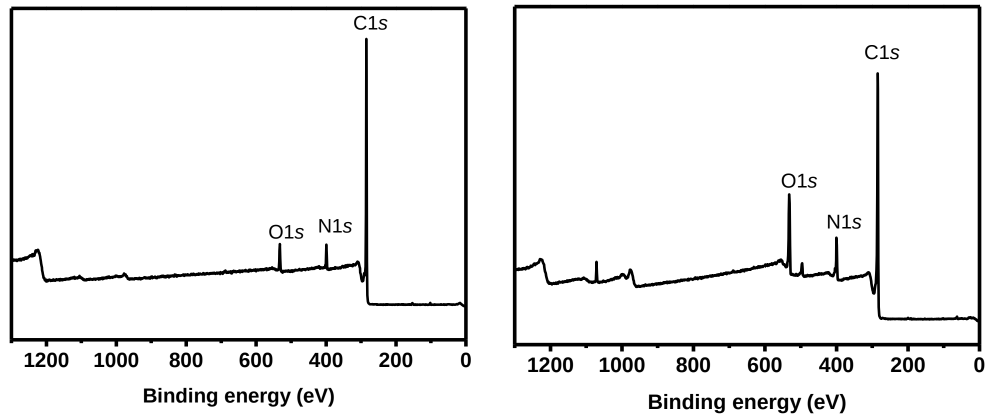

Supplementary Figure 24. XPS survey of **Im-COF-2** (left) and **Azo-COF-2** (right).

Supplementary Section 9. N_2 sorption isotherm experiments

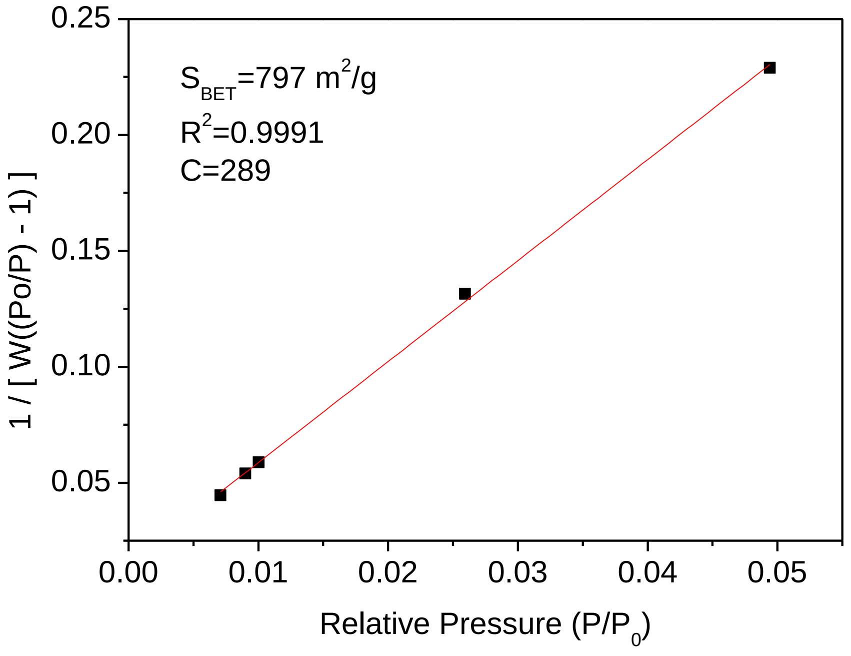

Supplementary Figure 25. BET surface area plot for **Im-COF-1**.

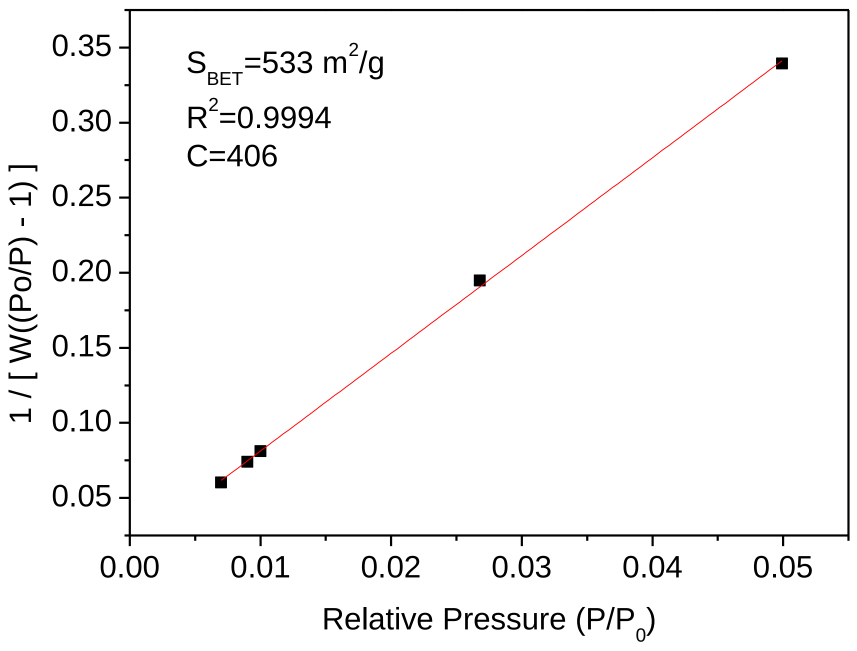

Supplementary Figure 26. BET surface area plot for **Azo-COF-1**.

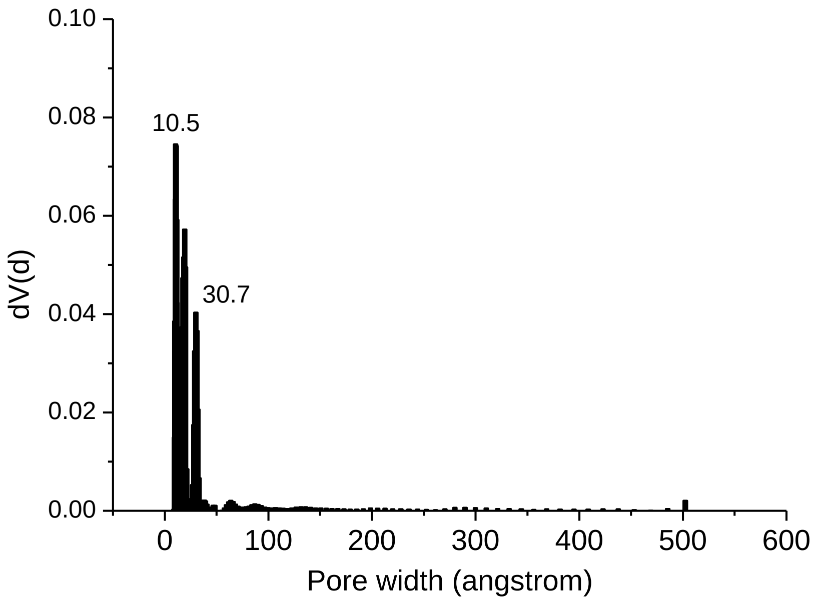

Supplementary Figure 27. Pore size distribution of **Im-COF-1**.

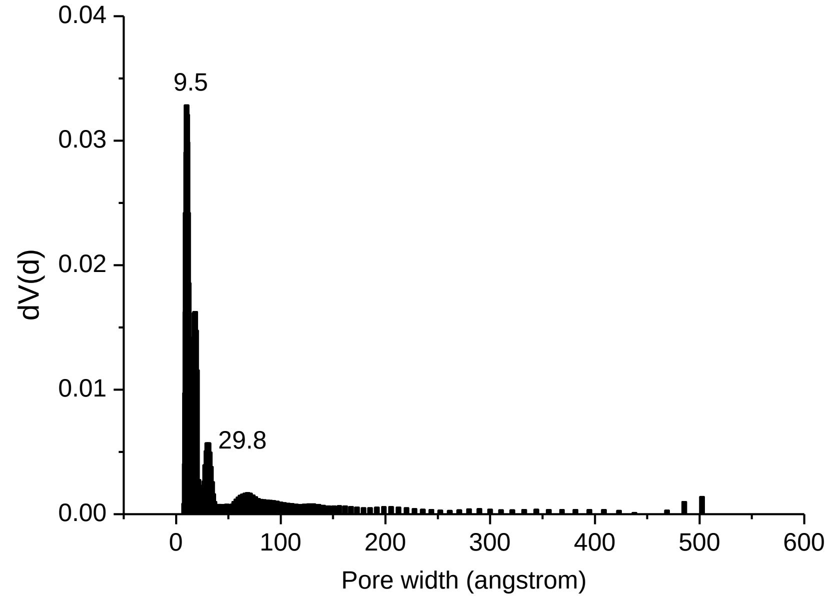

Supplementary Figure 28. Pore size distribution of **Azo-COF-1**.

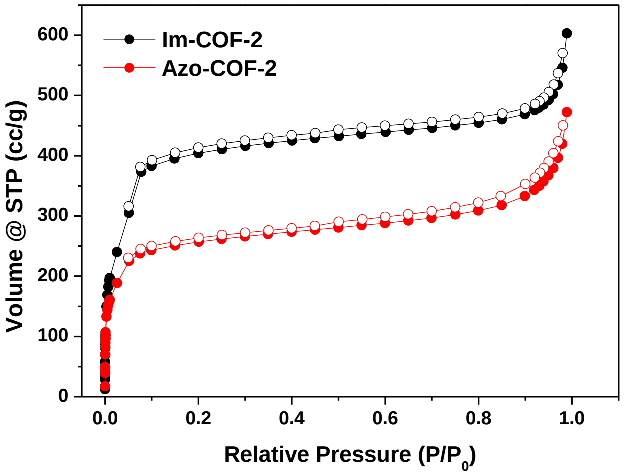

Supplementary Figure 29. Comparison of N_2 sorption isotherm between **Im-COF-2** and **Azo-COF-2**.

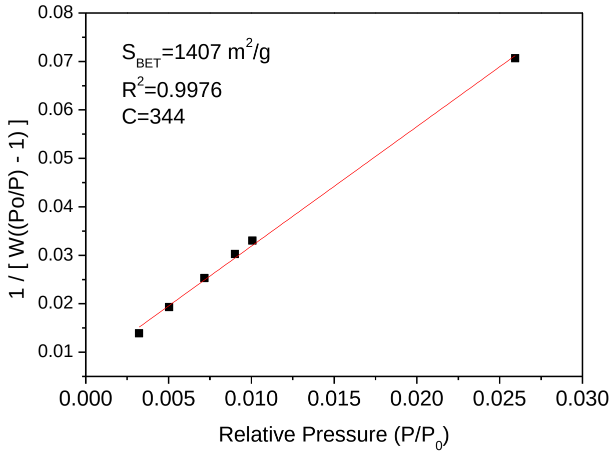

Supplementary Figure 30. BET surface area plot for **Im-COF-2**.

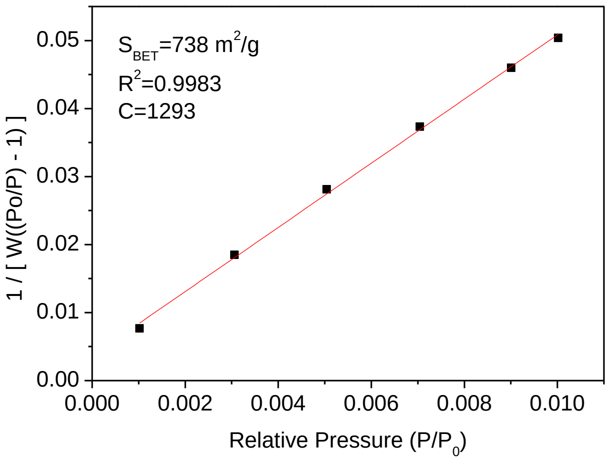

Supplementary Figure 31. BET surface area plot for **Azo-COF-2**.

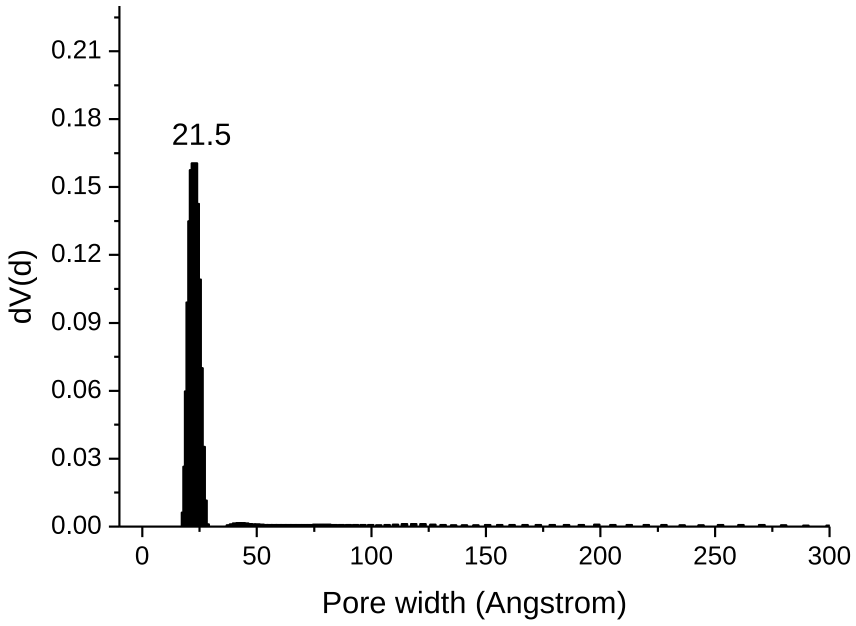

Supplementary Figure 32. Pore size distribution of **Im-COF-2**.

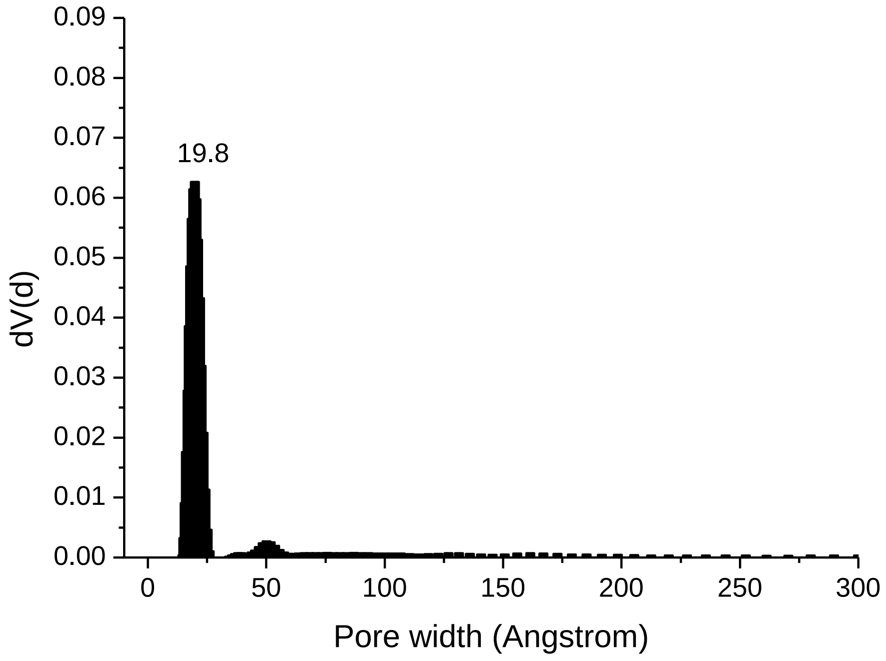

Supplementary Figure 33. Pore size distribution of **Azo-COF-2**.

Supplementary Section 10. SEM images

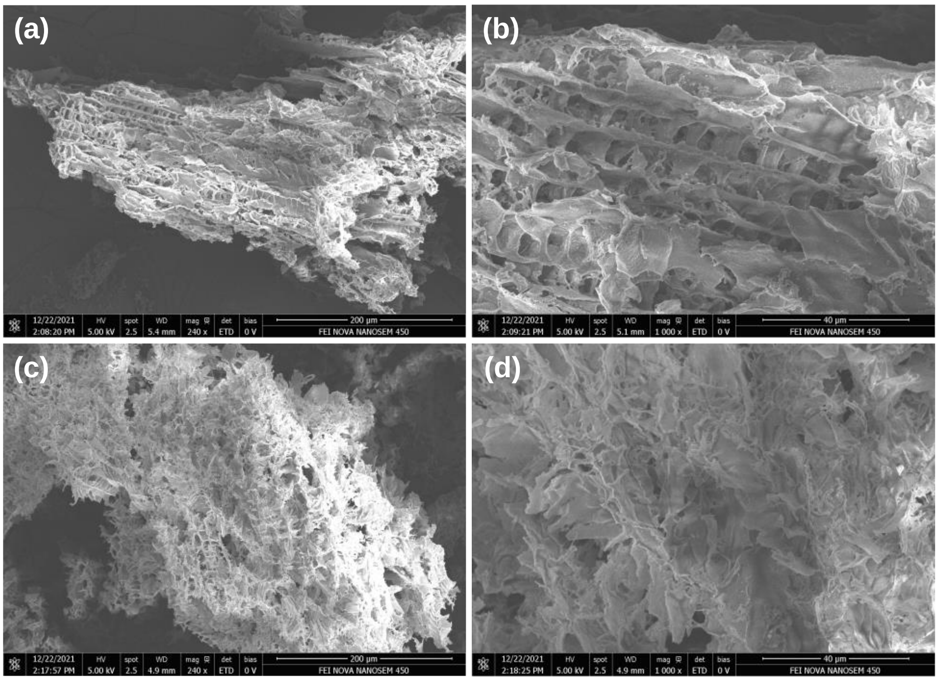

Supplementary Figure 34. SEM images with different scales: Im-COF-1: (a) 200 um, (b) 40 um. Azo-COF-1: (c) 200 um, (d) 40 um.

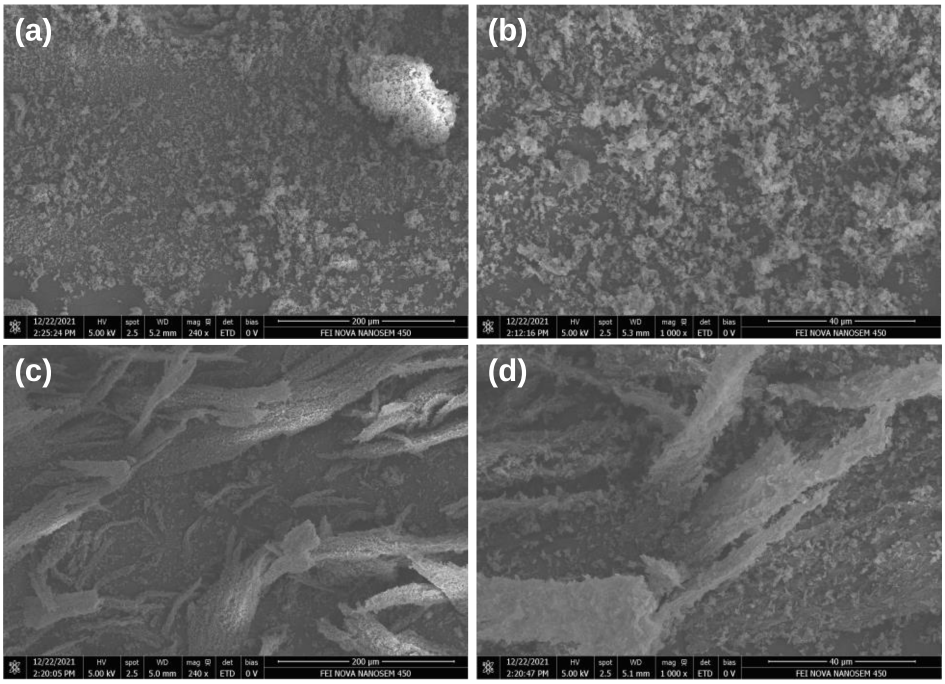

Supplementary Figure 35. SEM images with different scales: Im-COF-2: (**a**) 200 um, (**b**) 40 um. Azo-COF-2: (**c**) 200 um, (**d**) 40 um.

Supplementary Section 11. Hydrolysis experiments

Procedure for the hydrolysis experiments:

The hydrolysis experiments were performed under acidic condition following the procedure below. To a vial charged with a mixture of the COF sample (10 mg) and DMSO-d_6 (1 mL), DCl (0.1 mL, 12 M in D_2O) was added. The mixture was stirred at 60 ºC for 6 hours and the resulting mixture was subjected to ^{1}H NMR analysis after removing solid. It is worth to note that **Im-COF-1** is vulnerable to acid and thus heating is no need for its hydrolysis.

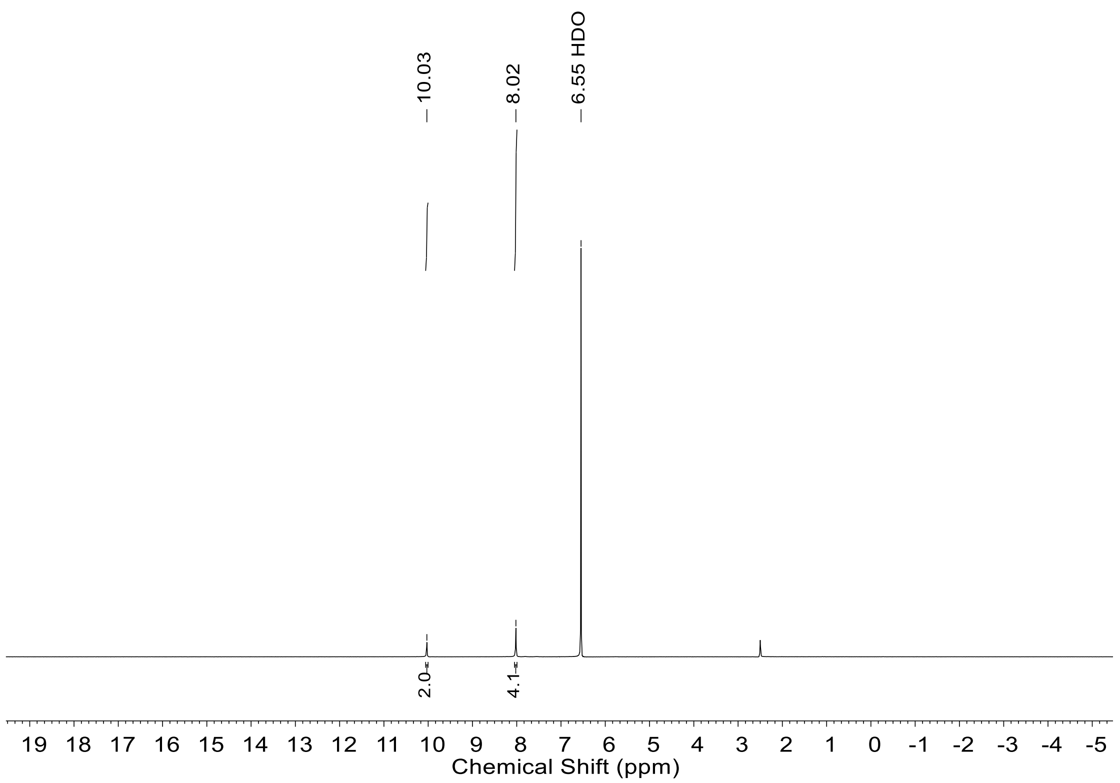

Supplementary Figure 36. ^{1}H NMR spectrum (400 M, DMSO-d_6) of **TPA** mixed with DCl.

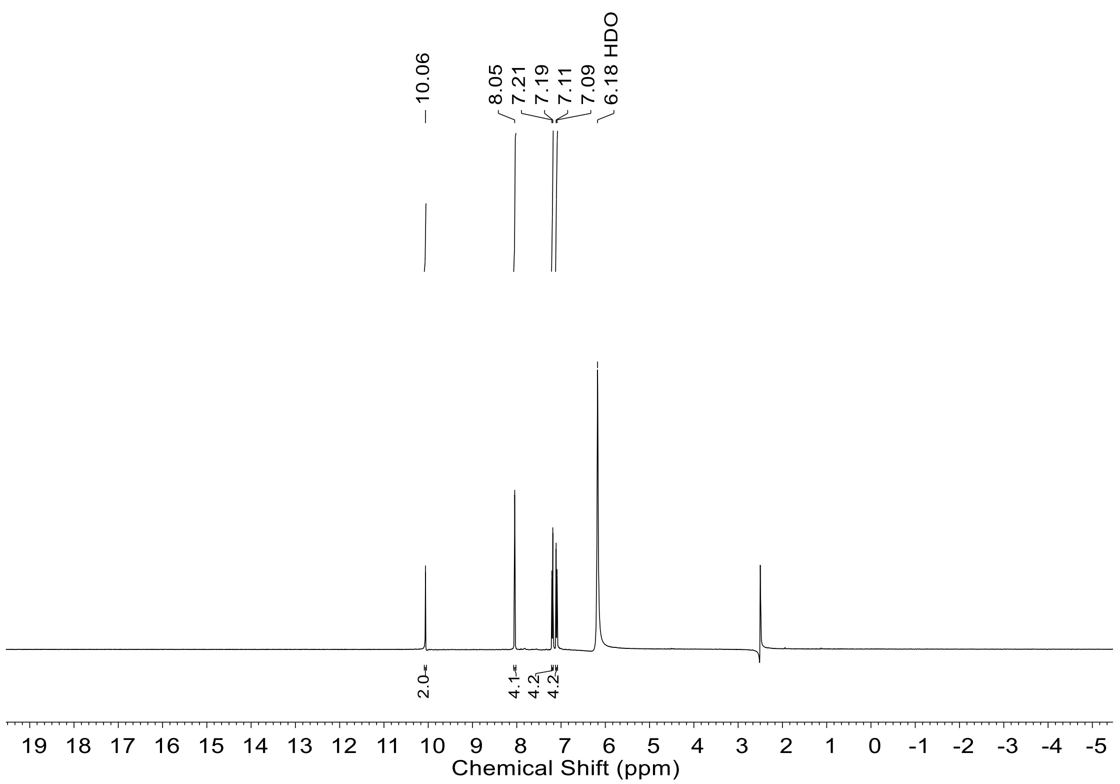

Supplementary Figure 37. Full ^{1}H NMR spectrum (400 M, DMSO-d_6) of the solution resulting from the hydrolyzation of **Im-COF-1**.

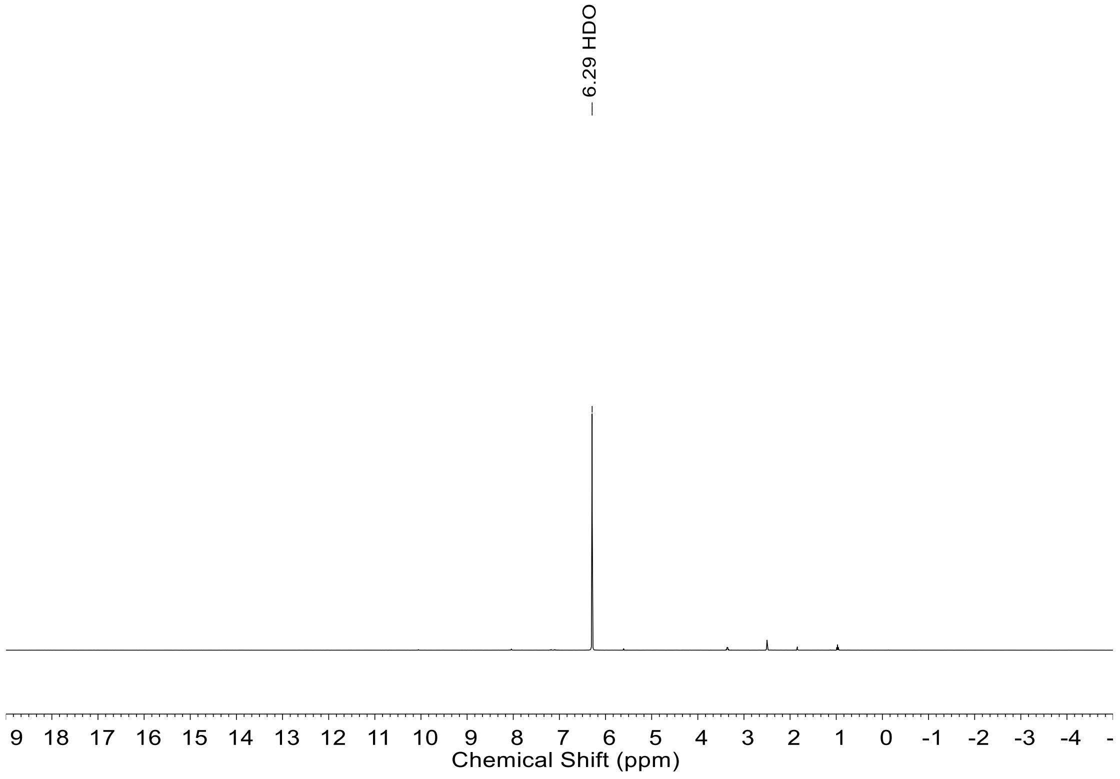

Supplementary Figure 38. Full ^{1}H NMR spectrum (400 M, DMSO-d_6) of the mixture resulting from the hydrolyzation of **Azo-COF-1**.

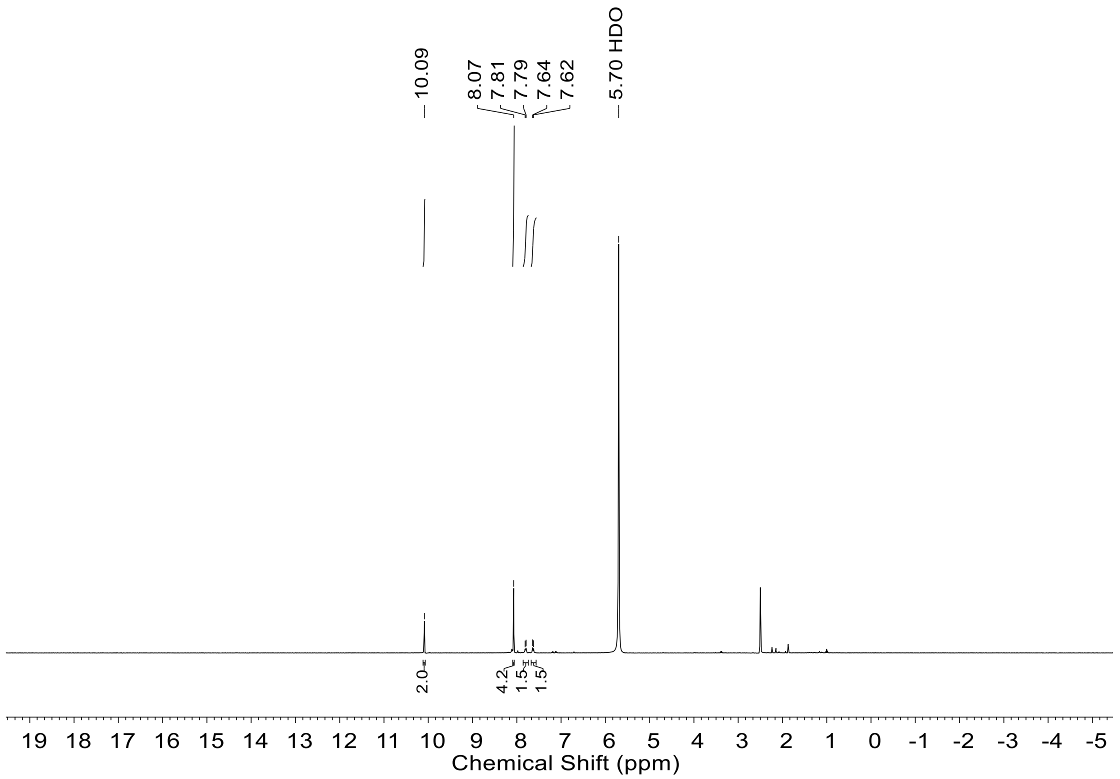

Supplementary Figure 39. ^{1}H NMR spectrum (400 M, DMSO-d_6) of the mixture resulting from the hydrolyzation of **Im-COF-2**.

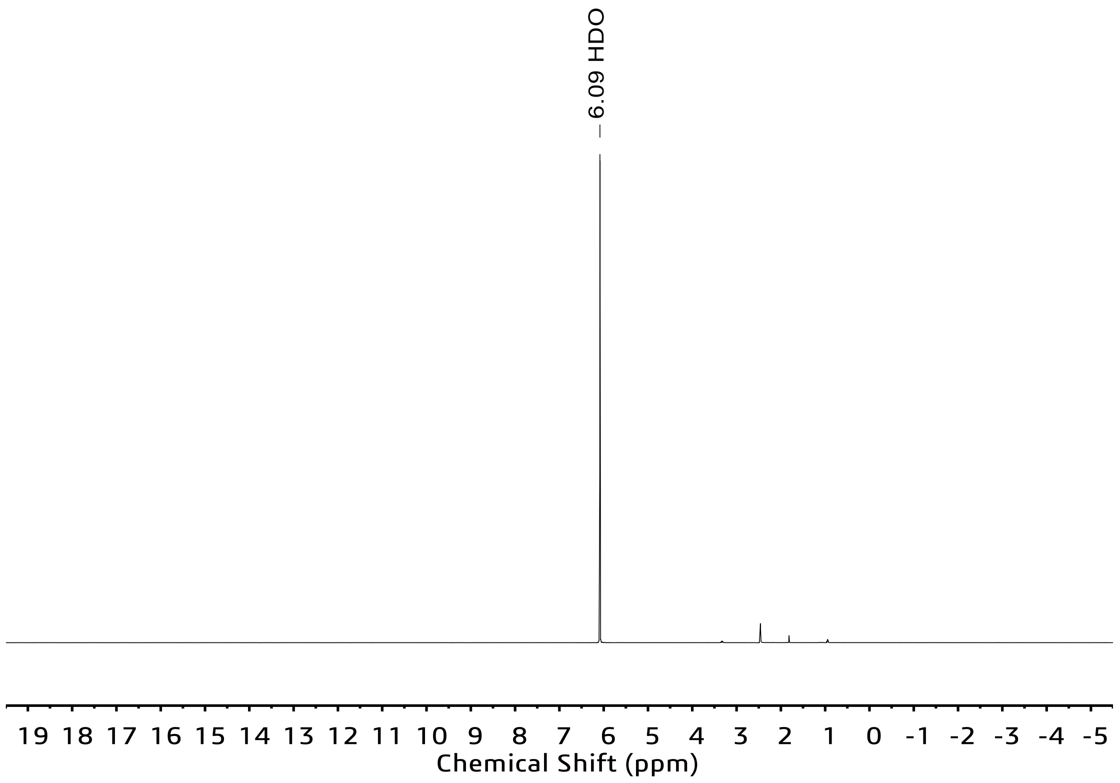

Supplementary Figure 40. ^{1}H NMR spectrum (400 M, DMSO-d_6) of the mixture resulting from the hydrolyzation of **Azo-COF-2**.

Supplementary Section 12. Solid-state UV-vis-NIR DRS

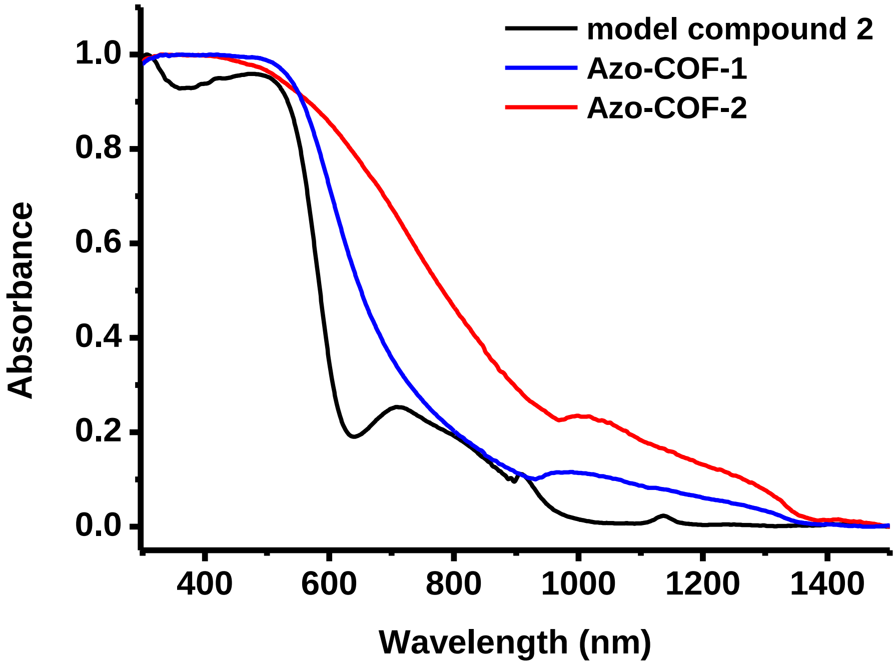

Supplementary Figure 41. Comparison of solid-state UV-vis-NIR DRS between model compound **2**, **Azo-COF-1**, and **Azo-COF-2**.

Supplementary Section 13. Photocatalytic degradation study

Procedure for photocatalytic degradation experiments:

The photocatalytic activity of the Azo-COF was evaluated by the degradation of Rh B under visible light irradiation. In a typical photocatalytic procedure, the photocatalyst (**Azo-COF-1**, 10 mg) was dispersed in an aqueous solution of Rh B (25 ppm, 100 mL), followed by magnetically stirring in dark. After reaching adsorption-desorption equilibrium, the system was exposed to a 300 W xenon lamp with an optical cutoff filter ($\lambda \geqslant 420$ nm). For the collection of UV-vis spectra, 1 mL of aliquot was extracted and centrifuged to remove the photocatalyst at the given intervals. After that, the resulting supernatant was diluted with distilled water (2.0 ml), and then analyzed with a UV–vis spectrophotometer. For comparison, the photocatalytic activity of **Im-COF-1** was also assessed following the similar experimental procedure.

The performance of photocatalytic degradation of Rh B catalyzed by the COFs was evaluated by plot of C/C_e versus time, where C_e and C are the concentrations of Rh B at the adsorption-desorption equilibrium and t minutes of the photocatalytic reaction, respectively. The concentration of Rh B is calculated based on the absorbance of the peak at 553 nm.

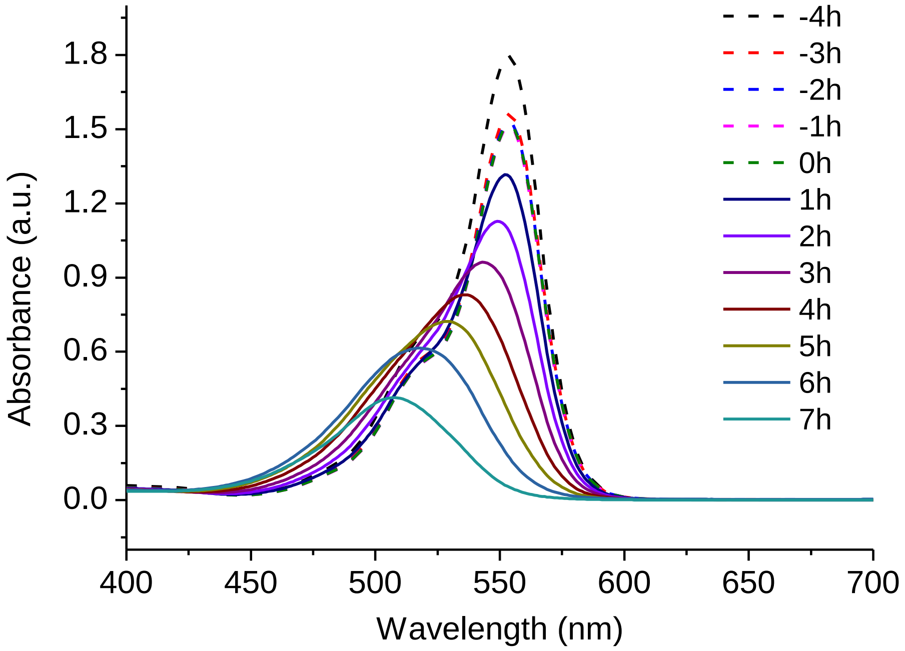

Supplementary Figure 42. UV-vis spectra recorded for photodegradation of Rh B (25 ppm) catalyzed by **Azo-COF-1**. Note: The region of -4h–0h is corresponding to the adsorption process in dark.

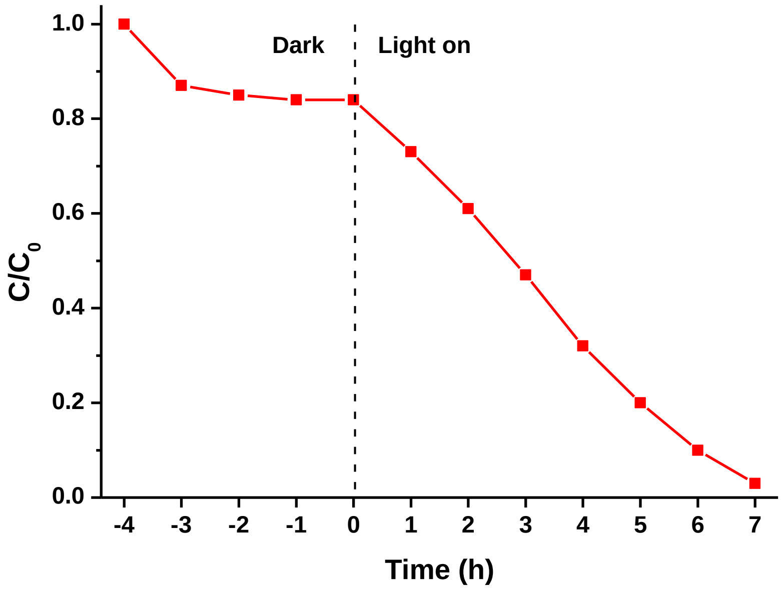

Supplementary Figure 43. Photocatalytic degradation of Rh B (25 ppm) catalyzed by **Azo-COF-1**. Note: C_0 refers to the initial concentration of Rh B before the adsorption in dark.

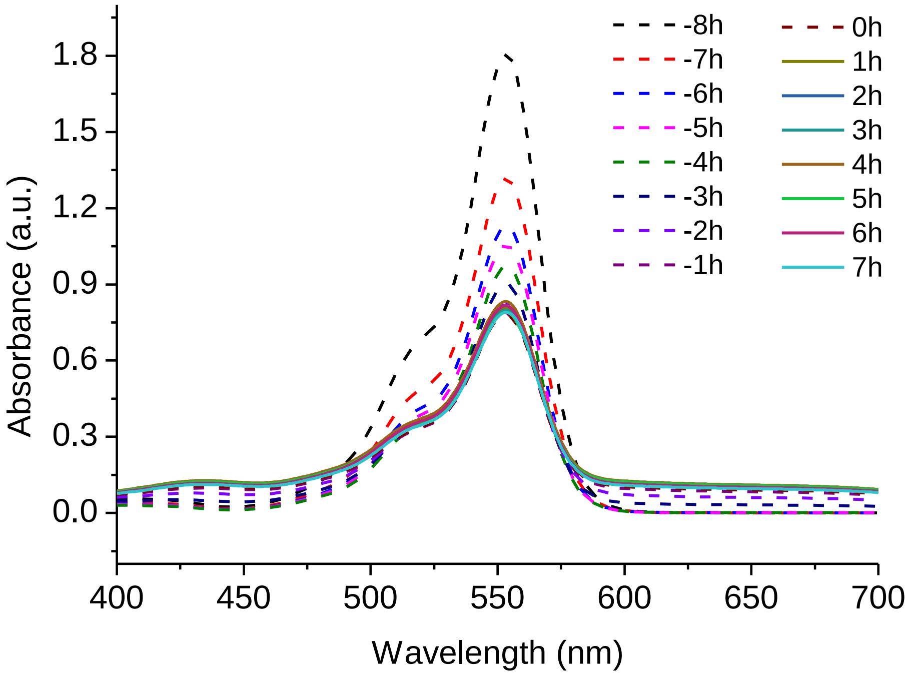

Supplementary Figure 44. UV-vis spectra recorded for photodegradation of Rh B (25 ppm) catalyzed by **Im-COF-1**. Note: The region of -8h–0h is corresponding to the adsorption process in dark.

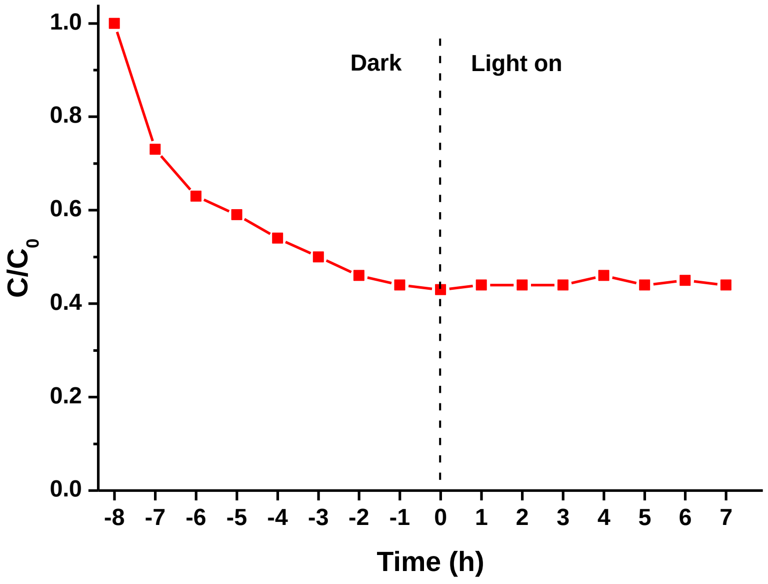

Supplementary Figure 45. Photocatalytic degradation of Rh B (25 ppm) catalyzed by **Im-COF-1**. Note: Note: C_0 refers to the initial concentration of Rh B before the adsorption in dark.

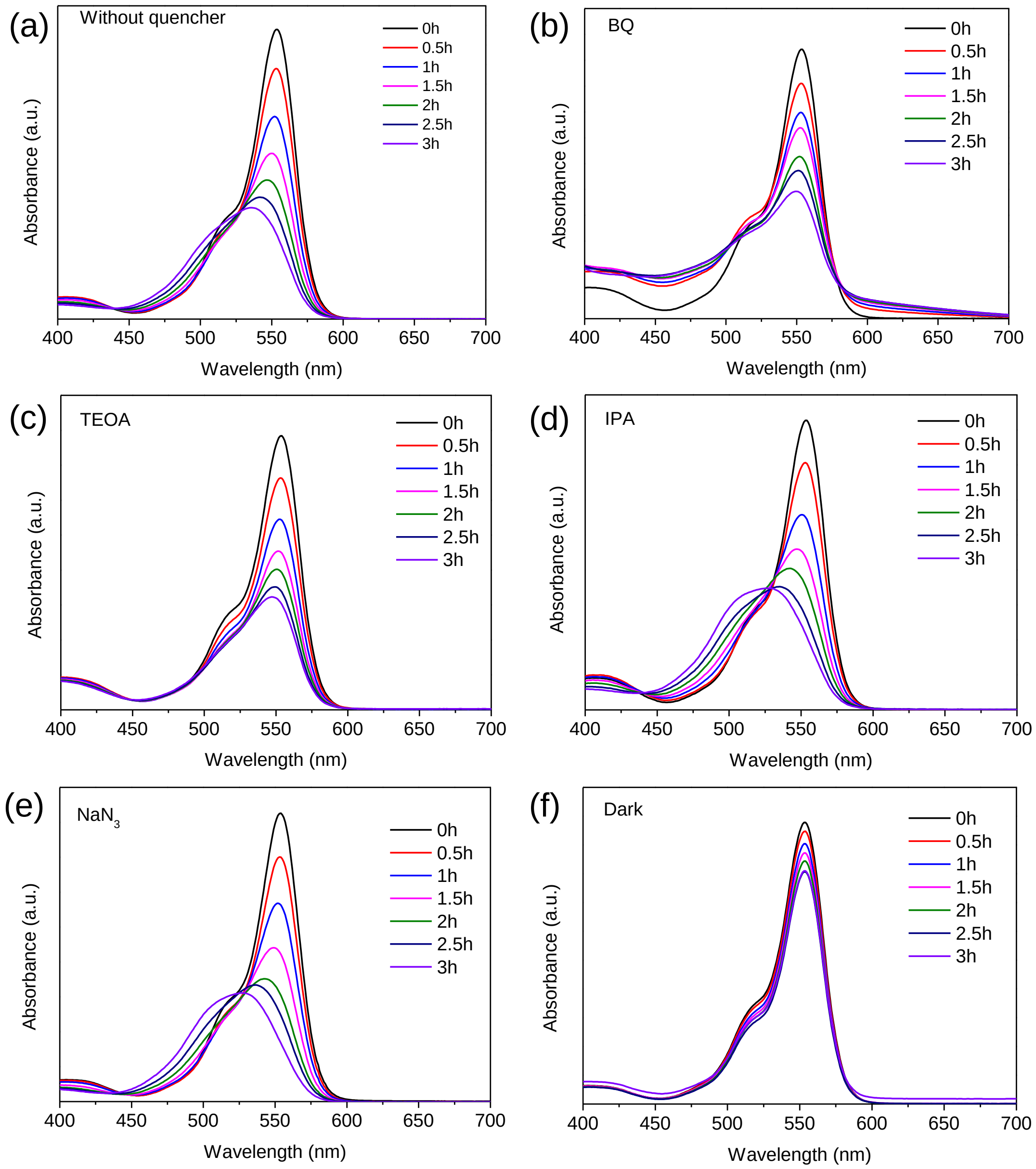

Supplementary Figure 46. UV-vis spectra recorded for photodegradation of Rh B (15 ppm) catalyzed by **Azo-COF-1** (5 mg) under different condition: (**a**) without the scavenger; (**b**) with $^{\cdot}O_2^{-}$ scavenger (p-benzoquinon, 0.1 mM); (**c**) with the hole scavenger (TEOA, 20 mM); (**d**) with ·OH scavenger (isopropanol, 0.1 mM); (**e**) with $^1O_2$ scavenger (NaN_3, 1.8 mM); (**f**) without the irradiation.

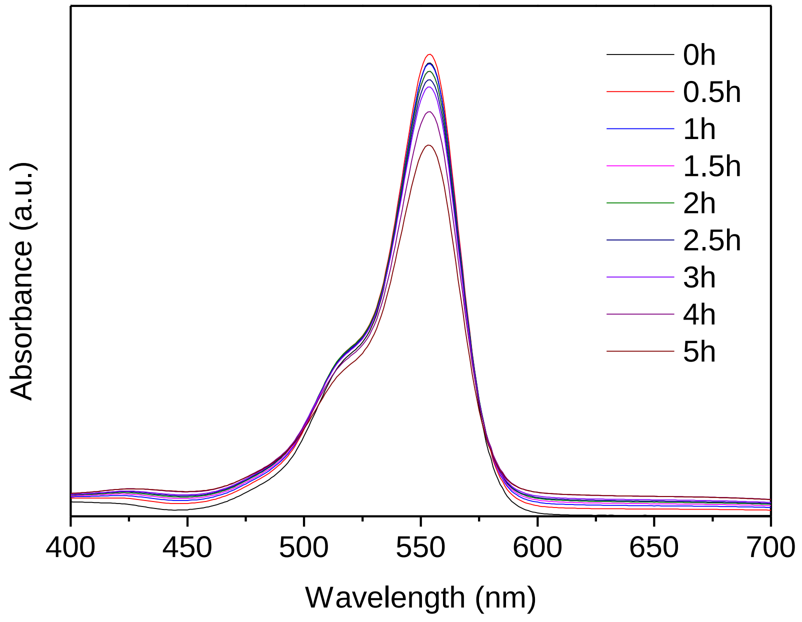

Supplementary Figure 47. UV-vis spectra recorded for photodegradation of Rh B (25 ppm) catalyzed by **Im-COF-1** (10 mg) without and with the presence of EDTA·2Na. Note: 336 mg EDTA·2Na was added into the reaction system after the irradiation of 3 hours.

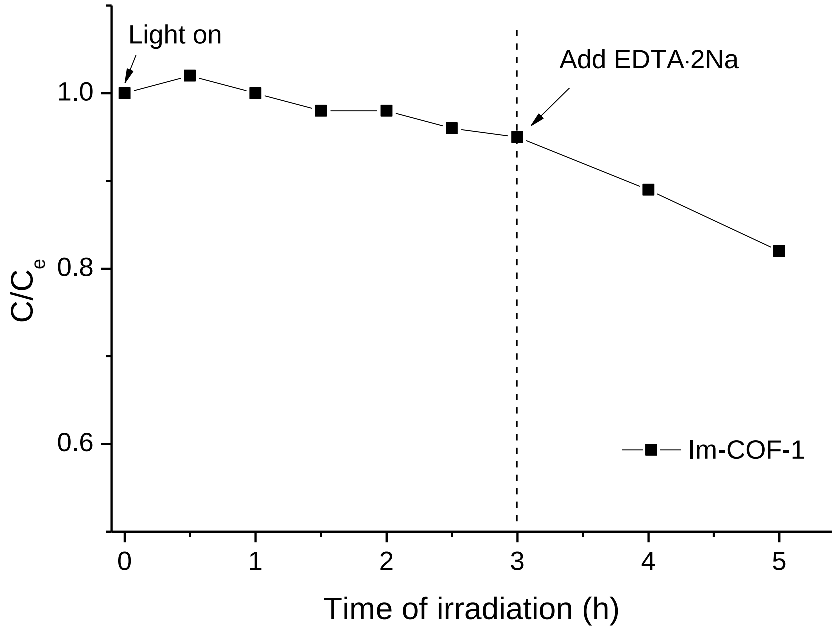

Supplementary Figure 48. Photocatalytic degradation of Rh B (25 ppm) catalyzed by **Im-COF-1** (10 mg) without and with the presence of EDTA·2Na. Note: 336 mg EDTA·2Na was added into the reaction system after the irradiation of 3 hours.

Supplementary Section 14. Fractional atomic coordinates of COFs

Supplementary Table 1. Fractional atomic coordinates for the unit cell of **Im-COF-1** with AA stacking.

P6							
a = b = 38.64347 Å, c = 4.98216 Å, α = β = 90 º, γ = 120 º							
C1	3.77395	-0.79288	0.65398	C20	3.56571	-0.93139	0.30456
C2	3.80762	-0.75569	0.59332	N21	3.66974	-0.85839	0.43978
C3	3.80685	-0.7326	0.37609	C22	3.7048	-0.84452	0.57143
C4	3.77178	-0.74755	0.22044	C23	3.73842	-0.80769	0.50212
C5	3.73798	-0.7845	0.28344	H24	3.77556	-0.80888	0.81254
C6	2.00063	-1.5184	0.48144	H25	3.83275	-0.74562	0.70872
C7	1.96531	-1.55666	0.48209	H26	3.77045	-0.73151	0.06097
C8	1.93522	-1.56692	0.67641	H27	3.71299	-0.79427	0.167
C9	1.90072	-1.60488	0.68424	H28	1.93848	-1.54639	0.81375
C10	1.89521	-1.63401	0.49366	H29	1.87948	-1.61121	0.82836
C11	1.92489	-1.6246	0.29736	H30	1.92163	-1.64558	0.15913
C12	1.95941	-1.58653	0.29315	H31	1.98048	-1.58079	0.15093
N13	1.8617	-1.67	0.5128	H32	1.84676	-1.6871	0.11716
C14	1.84005	-1.6954	0.31402	H33	3.58374	-0.97982	0.78947
C15	3.55686	-0.96323	0.48218	H34	3.64771	-0.92093	0.78003
C16	3.58817	-0.95799	0.65669	H35	3.60857	-0.87444	0.16204
C17	3.62611	-0.92329	0.65098	H36	3.54447	-0.93321	0.1792
C18	3.63421	-0.89235	0.46746	H37	3.70703	-0.86086	0.72501
C19	3.60348	-0.89665	0.29557				

Supplementary Table 2. Fractional atomic coordinates for the unit cell of **Azo-COF-1** with AA stacking.

P6							
a = b = 38.35857 Å, c = 4.57069 Å, α = β = 90 º, γ = 120 º							
C1	3.77041	-0.79711	0.42845	C19	3.59607	-0.89466	0.67197
C2	3.80332	-0.75849	0.43559	C20	3.56051	-0.93067	0.6378
C3	3.79847	-0.72474	0.47059	N21	3.66601	-0.84952	0.58249
C4	3.75914	-0.73164	0.50163	N22	3.69887	-0.84469	0.48506
C5	3.72649	-0.77034	0.51245	C23	3.73125	-0.80411	0.47448
C6	2.00124	-1.51744	0.4001	H24	3.77521	-0.82282	0.39873
C7	1.96482	-1.55993	0.40862	H25	3.83373	-0.75406	0.40929
C8	1.92716	-1.57033	0.2861	H26	3.75433	-0.70594	0.53142
C9	1.89429	-1.60851	0.32182	H27	3.69624	-0.77472	0.54782
C10	1.89766	-1.63906	0.46486	H28	1.92163	-1.54854	0.1717
C11	1.93572	-1.62949	0.56993	H29	1.86493	-1.61521	0.23386
C12	1.96818	-1.59083	0.54977	H30	1.9393	-1.65262	0.68527
N13	1.86526	-1.67872	0.51893	H31	1.99632	-1.5853	0.65362
N14	1.83034	-1.68404	0.48404	H32	3.59076	-0.97237	0.10429
C15	3.55725	-0.95899	0.42965	H33	3.65449	-0.90981	0.18685
C16	3.59162	-0.95079	0.26617	H34	3.59766	-0.87275	0.83387
C17	3.62775	-0.91559	0.31329	H35	3.53452	-0.93673	0.77276
C18	3.63072	-0.88657	0.5152				

Supplementary Table 3. Fractional atomic coordinates for the unit cell of **Im-COF-2** with AA stacking.

C2/M			
a = 35.63423 Å, b = 31.65881 Å, c = 3.99382 Å, α = β = γ = 90 º			
C1	0.03281	0.90997	0.59855
C2	0.03132	0.95474	0.62782
C3	0.0604	0.97801	0.78023
C4	0.27738	0.71745	0.49466
C5	0.28425	0.75649	0.33418
C6	0.24275	0.71137	0.65996
C7	0.30405	0.68509	0.49882
N8	0.33766	0.68962	0.34707
C9	0.43391	0.61389	0.35926
C10	0.43284	0.65268	0.18285
C11	0.39993	0.67728	0.17781
C12	0.36732	0.66334	0.34356
C13	0.36765	0.62379	0.50529
C14	0.40059	0.59995	0.51643
H15	0.08235	0.96332	0.89323
H16	0.30877	0.762	0.20844
H17	0.23683	0.68377	0.78234
H18	0.29769	0.65797	0.62498
H19	0.45605	0.66344	0.05855
H20	0.3998	0.70562	0.05426
H21	0.34448	0.61216	0.62297
H22	0.39994	0.57231	0.64645
C23	0	0.11156	0.5
C24	0	0.97741	0.5
H25	0	0.14387	0.5

Supplementary Table 4. Fractional atomic coordinates for the unit cell of **Azo-COF-2** with AA stacking.

C2/M			
a = 35.51294 Å, b = 31.54341 Å, c = 3.98441 Å, α = β = γ = 90 º			
C1	0.03318	0.90942	0.59843
C2	0.03188	0.95446	0.62227
C3	0.06153	0.97786	0.76822
C4	0.28062	0.72137	0.47063
C5	0.28304	0.76254	0.33307
C6	0.24739	0.70906	0.6374
N7	0.3089	0.69292	0.45402
N8	0.34129	0.69831	0.30877
C9	0.43405	0.61567	0.34926
C10	0.43509	0.65402	0.16352
C11	0.40354	0.68102	0.14834
C12	0.36986	0.67009	0.31247
C13	0.36781	0.63099	0.48296
C14	0.39954	0.60449	0.504
H15	0.08373	0.96298	0.87953
H16	0.30698	0.77175	0.21072
H17	0.24544	0.67938	0.73938
H18	0.45902	0.66286	0.03934
H19	0.40508	0.7089	0.01804
H20	0.34338	0.62233	0.59983
H21	0.39732	0.57729	0.64161
C22	0	0.11185	0.5
C23	0	0.97727	0.5
H24	0	0.14427	0.5

Supplementary References

1. Ermakov, O. A. & Komkova, Y. F. *J. Org. Chem. USSR (Engl. Transl.)* **20**, 2053-2054 (1984).

2. Zhu, K. et al. *Nat. Chem.* **7**, 514-519 (2015).
